# Integrating physical modeling with artificial intelligence for predicting fish survival zones in polluted rivers to maintain a sustainable aquaculture industry

**DOI:** 10.1038/s41598-026-57547-6

**Published:** 2026-06-18

**Authors:** Hussein Karam Abd El-Sattar, Mohammed Elshambakey, Ahmed Saleh, Samar Antar

**Affiliations:** 1 Faculty of Science, Mathematics Department, Computer Science Division, Ain Shams University, Abbassia, 11566 Cairo Egypt; 2 Knowledge-Based Systems and Robotics Dept, Informatics Research Institute, City of Scientific Research and Technological Applications, New Borg El-Arab, Alexandria Egypt; 3 Department of Basic Science, Egyptian Academy for Engineering and Advanced Technology, Cairo, Egypt; 4 Department of Artificial Intelligence, Faculty of Computers and Artificial Intelligence, Hurghada University, Hurghada, Egypt

**Keywords:** Artificial intelligence (AI), Physical AI, Random forests, Multi-layer perceptron, Water research, Pollution–aeration equations, Machine and deep learning algorithms, Ecology, Ecology, Environmental sciences, Mathematics and computing

## Abstract

Water quality prediction and management are crucial for ensuring the sustainability of water supplies. Contaminated water can harm humans and aquatic life. As the demand for seafood grows, the aquaculture industry faces several obstacles, including disease management, feeding optimization, water quality monitoring, and aquaculture area extraction. Recently, aquaculture systems have increasingly used AI techniques to successfully and sustainably handle these issues. However, traditional AI techniques such as random forest (RF) and multi-layer perceptron (MLP) among others frequently face data scarcity and poor physical consistency. This research bridges this gap by integrating physical sciences with AI algorithms through the solution of the two coupled pollution–aeration equations to generate a high-fidelity physics-derived dataset of 50,000 observations over an extended spatial domain ranging from 0 to 4. This dataset is then used to train a novel hybrid RF–MLP algorithm to identify fish-survival zones within a polluted river at a given time, while determining the minimum allowable water velocity and the upstream dissolved oxygen level required to maintain environmentally safe conditions along the entire river reach. The proposed algorithm employs a three-stage sequential residual learning logic, combining RF’s stable feature partitioning with MLP’s improved non-linear error correction. The algorithm’s performance was benchmarked against nine standalone AI algorithms using a comprehensive suite of metrics. The experiments demonstrated exceptional precision with a Correlation Coefficient (CC) of 0.9999999973, a Scatter Index (SI) of 0.00007326, a Willmott’s Index (WI) of 0.9999999986, a Test RMSE of 0.00012966, and a 0.9999999692. Beyond accuracy, the hybrid algorithm demonstrated superior computational efficiency, training in just 22.58 s—a 24.45-fold reduction compared to BiLSTM architectures. These results provide a robust tool for decision-makers to identify optimal river reaches for fish farms based on minimum water velocity and permissible dissolved oxygen transfer levels, bridging the gap between theoretical physics and industrial aquaculture management.

## Introduction

Aquaculture industry provides an abundance supply of fish resources for humanity. The aquaculture industry faces numerous challenges; particularly water quality (WQ) monitoring. Monitoring and predicting water quality is crucial for identifying pollution and environmental concerns, as well as for making educated decisions and managing water resources sustainably^1^. The discharge of industrial waste into water resources, such as rivers and lakes, has increased as industrial processes have expanded and innovated. Such discharge leads to the deterioration of water quality, which directly affects human lives, fish survival, and environmental health in general^2^. As the demand for fish as a food source increased, the aquaculture environment steadily developed to provide fish for human consumption. Every year, there are tons of fish mortalities due to water contamination. Fish mortality is an essential production and welfare metric in the aquaculture industry. To address the issue of fish mortality, water quality (WQ) monitoring is critical to make aquaculture production profitable and sustainable^3,4^. Several factors influence WQ, including fish density, feed quality, feeding interval, climate, and water quality parameters. Water temperature (WT), free ammonia (AMM), fecal coliform (TC), fecal coliform (FC), conductivity (COND), biochemical oxygen demand, water acidity/base level (pH), and dissolved oxygen (DO), among others, are critical water quality parameters in aquaculture. Meteorological conditions and the intricate interdependence relationships between different water quality parameters all have an impact on aquaculture water quality. As a result, changes in water quality parameters exhibit nonlinear properties, resulting in low prediction accuracy. According to^5,6^, accurate dissolved oxygen (DO) concentration prediction is an important aspect of water quality monitoring and assessment, as it provides essential information about the chemical, physical, and biological characteristics of water bodies, which is critical for ensuring the healthy growth of aquatic species. An adequate DO concentration (typically above 5.0 mg/L for most freshwater fish species) can support normal growth and development, whereas low DO concentrations (typically below 3.0 mg/L) may inhibit biological activity, leading to significant economic losses and even fish mortality^7^. Scholars worldwide have employed several methods, including the Markov model, Grey model, support vector regression (SVR), autoregressive integrated moving average (ARIMA), and seasonal autoregressive integrated moving average model (SARIMA), among others, to estimate DO concentrations^8–10^. Because the water quality parameters are nonlinear, dynamic, variable, and complicated, these techniques often struggle to capture the complex and nonlinear hydroclimatic processes and nonstationary patterns that dynamically vary over space and time^11,12^. These issues highlight the need for innovative technologies such as artificial intelligence (AI), including machine learning and deep learning, to improve the accuracy and interpretability of water quality prediction, as well as optimize commercial aquaculture practices, efficiency, productivity, and profitability, which is the primary goal of this research.

### Motivations

As the demand for fish as a food source grew, the aquaculture environment evolved to offer fish for human consumption. The aquaculture business has various obstacles, most notably water quality (WQ) monitoring. To ensure sustainability while optimizing efficiency, productivity, and profitability, the aquaculture industry has turned to AI techniques such as random forest (RF), multi-layer perceptron (MLP), Support Vector Machine (SVM), and Long short-term memory (LSTM), among others, as an aid in water quality monitoring and prediction^13–21^. Because AI techniques can efficiently solve complicated nonlinear problems, particularly those related to water quality monitoring and prediction, they have recently become a global trend in sustainable development research^22^. However, most AI algorithms require massive amounts of datasets to achieve higher accuracies, which are determined using costly and time-consuming lab and statistical analyses that require sample collection, transportation to labs, and a significant amount of time and calculation, all of which are inefficient. Integrating physical sciences with advanced AI algorithms has recently emerged as an effective way to address the training data shortage, increase model generalizability, and provide a robust solution for predicting water pollution concentration and management^23–29^. Physics-based knowledge is often presented in two ways: One is to use physics-based equations that typically establish a precise relationship between certain inputs and outputs. Another one is to employ numerical models used to simulate complex systems. Motivated by these considerations, we propose a new physics-guided hybrid AI algorithm that combines physics-based models, such as the two coupled pollution-aeration equations (PAEs), with AI algorithms for monitoring suitable areas for fish survival in a polluted river at a particular time along the river with the minimum permissible water velocity and the minimum permissible oxygen transfer from air to water. The advection-diffusion equation (ADE) is commonly used for predicting water pollution concentrations. The aeration equation, which is made up of two coupled non-linear partial differential equations (PDEs) and their associated conditions, is a strong instrument for imposing constraints on urban and farming practices. The coupling happens when oxygen combines with pollutants produced in industrial and agricultural areas, resulting in innocuous chemicals. The pollution-aeration model’s analytical and numerical solutions allow for the assessment of fish survival scenarios, which typically demand a dissolved oxygen concentration greater than 30% of saturated dissolved oxygen concentration^30^.

### Contributions

The primary contributions of this research are summarized as follows:

- **Advancement of Physics-AI Integration in Hydrology**: Expanding on recent research by^23–29^, this study establishes a synergistic relationship between relevant physical sciences and AI. The methodology serves as a reproducible framework for water research, empowering researchers to bridge the gap between deterministic physical models and data-driven architectures in diverse environmental contexts.
- **Novel Heterogeneous Boosting physics-guided hybrid (RF-MLP) Algorithm**: A novel physics-guided hybrid RF-MLP algorithm that integrates a physics-based model with AI algorithms to improve performance and accuracy for predicting both pollution concentration and dissolved oxygen, particularly when data scarcity and quality are the key factors. The proposed algorithm employs a three-stage sequential residual learning logic, combining RF’s stable feature partitioning with MLP’s improved non-linear error correction. Unlike existing hybrid AI implementations that rely on meta-heuristic optimization or simple ensemble, the novelty of the proposed RF-MLP lies in its heterogeneous boosting architecture. It specifically utilizes a sequential residual learning logic to bridge the gap between discrete tree-based partitioning and continuous physical transport phenomena.
- **Synthetic Data Generation via Coupled Equations**: To address the common challenge of data scarcity and the high cost of field collection in water research, this study utilizes the solution of two coupled pollution and aeration equations to generate a high-fidelity dataset of 50,000 spatial observations. This approach ensures physical consistency and provides a robust foundation for training complex machine learning algorithms over an extended spatial domain ranging from 0 to 4.
- **Restoration of Physical Gradients**: The algorithm successfully resolves the “step-like” artifacts typical of tree-based models. By using the MLP as a universal function approximator for residuals, the hybrid algorithm restores the smooth exponential curvature and continuous gradients required for physical fidelity to the coupled transport process.
- **Computational Performance Optimization**: The research demonstrates a superior performance-per-second ratio. The proposed hybrid algorithm achieves an order-of-magnitude reduction in RMSE compared to deep learning peers like GRU and LSTM while requiring significantly less training time (22.58 s compared to 552.21 s for BiLSTM, representing an approximately 24.45-fold reduction in training duration).
- **Empirical Validation of Predictive Precision**: Through exhaustive comparative analysis, the proposed hybrid algorithm achieved near-perfect metrics, including a Test $\:{R}^{2}$ of 0.9999999692, a Correlation Coefficient (CC) of 0.9999999973, and a Willmott’s Index of Agreement (WI) of 0.9999999986, outperforming nine individual AI algorithms.
- **Sustainable Development Goals (SDG)**: It outperforms nine individual AI algorithms in terms of both predictive outcomes and training efficiency, assisting environmental decision-makers by contributing to Clean Water and Sanitation (SDG 6) and Good Health and Well-Being (SDG 3), among other goals.

## Related work

### Relevant research on the pollution-aeration equations (PAEs)

Both human life and aquatic life are at risk due to chemical and biological pollution contaminating rivers worldwide. Contaminant transport through river water under spatially and temporally varying flow conditions is crucial for many environmental, biophysical, and ecological applications, including pollutant treatment and water quality management. The current study investigates the remediation of a polluted river using dissolved oxygen (DO) enrichment through controlled clean-water release, with the aim of monitoring environmentally safe zones for fish survival at a given time. In addition, an AI-based framework is employed to predict the coupled dynamics of pollutant concentration and dissolved oxygen, enabling efficient identification of suitable habitats and supporting water-management decisions that maintain dissolved oxygen above the ecological threshold for aquatic life. The solution of the two coupled pollution and aeration equations for flow in a river is utilized to achieve this goal. The coupled pollution–aeration equations governing river flow are solved using a finite difference scheme. In addition, an analytical solution is employed under simplified conditions to validate the numerical results. This combined analytical–numerical framework enables an accurate and reliable investigation of pollutant transport and dissolved oxygen dynamics in polluted rivers under realistic environmental conditions. For instance, the authors of^31^ used the advection-diffusion equation (ADE) to characterize the dynamics of river pollution and used a simulation study to derive a numerical solution. By splitting the river into two regions^32^, investigated an analytical solution for a one-dimensional ADE of the pollutant concentration. The authors of^33^ addressed the space–time-dependent ADE in two-dimensional conservative solute transport in a heterogeneous porous medium for different input sources analytically^34^. Investigated the use of clean water to remediate contamination in a river. The authors of^35^ offered analytical and numerical solutions for pollution concentration with exponentially and evenly increasing sources. The authors of^36^ examined the numerical treatment of the mathematical model for water contamination; they solved the generalized transport equation using an implicit centred difference scheme in space and a forward difference scheme in time. A grey differential model was employed by^37^ to develop a numerical simulation of river water contamination. For the mathematical model of the transport equation in a straightforward rectangular box domain^38^, offered a numerical solution. The ADE with variable coefficients and for anisotropic media was solved by^39^ using the boundary element approach. A numerical simulation of one-dimensional ADE utilizing an explicit centered difference scheme and a Crank-Nicolson scheme for specified initial and boundary data was reported by^40^. The authors of^41,42^ developed a mathematical model to help understand the behavior of the pollution-aeration process, its relationship to injected contaminants along a river, and its effect on fish survival. The suggested approach helps regulate industrial, agricultural, and urban behaviours and imposes restrictions as necessary. Finally^43^, carried out a comprehensive study in the atmospheric science community by comparing the deep neural network (DNN) with the finite difference techniques (FDM) to solve the 2D advection-diffusion equation to characterize the long-range transport of atmospheric pollutants. The results show that the DNN solver is the most accurate, with the mean absolute error and maximum absolute error of fluid concentration reduced by up to two orders of magnitude when compared to the FDM-based method, corresponding to relative error reductions of 95% and 97%, respectively. For measuring the generality of neural network layers across a continuously-parameterized set of tasks^44^, proposed a method based on the singular vector canonical correlation analysis (SVCCA). They demonstrate this method by investigating generality in neural networks trained to solve parameterized boundary value issues using the Poisson partial differential equation^45^. used the Laplace transformation technique to derive an analytical solution for the unsteady-state aeration model in its linear form. The authors of^46^ developed a numerical solution for the non-linear aeration model in two case studies. Studies on modeling the relationship between fish survival and population dynamics have been conducted^47–50^. Experimentally, the study of aeration systems is a rich and active research subject. Researchers address several aeration technologies and their usefulness in the context of design^51–54^.

### Related research for AI approaches

Artificial intelligence (AI) approaches have recently been used more extensively in sustainable development research^22^ to describe solute movement in porous media, particularly in studies on river pollution and water quality prediction (e.g.^20,55^). Because of their inherent ability to manage enormous datasets, intricate variable interactions, and complex non-linear relationships, these approaches provide significant benefit over conventional statistical and mechanistic models. According to recent literature reviews^14–21^, building hybrid algorithms by combining different machine learning and deep learning algorithms has gained the attention of researchers, as it can overcome the limitations of individual algorithms and achieve higher performance and accuracy than individual ones. Some of the prior research in this area is described below.

- A hybrid deep learning (DL) model, CNN-LSTM and CNN-GRU, is presented in^56^ for aquaculture water quality prediction (WQP) by combining convolutional neural networks (CNN) with long short-term memory (LSTM) and gated recurrent units (GRU). Similarly, the authors of^17^ developed a hybrid model for WQP that combines CNN, LSTM, and a self-attention mechanism.
- For the objective of forecasting the river hydrodynamic changes^57^, presented a coupling framework of a hydrodynamic model and machine learning approach, named GRU-HD, for modeling lake-connected river systems. The framework is based on the integration of the Gated Recurrent Unit (GRU) with a 1-D HydroDynamic model (GRU-HD), taking into account the advantages of the interpretability of physical hydrodynamic modeling and the adaptability of machine learning.
- Reference^58^ introduced hybrid models that integrate temporal pattern attention (TPA) mechanisms with advanced neural networks, including feed-forward neural networks (FFNNs) and long short-term memory networks (LSTMs), for predicting water quality in complex systems.
- To predict pan evaporation (Ep), a critical factor in water resource management^59^, presented two hybrid models, called GA-RF and GA-MLP, for modelling Ep at a target station. These models use data from adjacent stations by combining two base models: Random Forest (RF) and Multi-Layer Perceptron (MLP), along with their optimized counterparts created through a Genetic Algorithm (GA).
- The authors of^60^ presented a hybrid Wavelet-CNN-LSTM model for short-term water demand forecasting that incorporates the time-frequency decomposition features of Wavelet Multi-Resolution Analysis (MRA) into CNN-LSTM, an advanced deep learning model. Experimental results show that, in both single-step and multi-step prediction, the Wavelet-CNN-LSTM model outperforms four distinct deep learning models: ANN, Conv1D, LSTM, and a gated recurrent unit (GRU).
- Reference^61^ proposed two hybrid decision tree-based machine learning models, called CEEMDAN-XGBoost and CEEMDAN-RF, for short-term water quality prediction. The two hybrid models’ basic components are random forest (RF) and extreme gradient boosting (XGBoost), each of which introduces a sophisticated method for denoising data: complete ensemble empirical mode decomposition with adaptive noise (CEEMDAN).
- The authors of^62^ studied hybrid models that combine machine learning (ML) with statistical techniques (such as principal component analysis (PCA)) to anticipate and classify water quality.
- The water quality class (WQC), a unique class defined on the basis of the water quality index (WQI), and the water quality index (WQI), a single index to describe the overall quality of water, are estimated by^63^ using a number of supervised machine learning algorithms. According to the experimental results, the most effective methods for predicting the WQI are gradient boosting (learning rate of 0.1) and polynomial regression (degree of 2), with mean absolute errors (MAE) of 1.9642 and 2.7273, respectively. On the other hand, the WQC is most effectively classified by the multi-layer perceptron (MLP), which has a configuration of (3, 7) and an accuracy of 0.8507.
- The authors of^64^ proposed a hybrid CNN-LSTM-based deep learning framework for predicting water level and quality. The authors of^65^ proposed a new hybrid deep learning model for air quality forecasting. One-dimensional Convolutional Neural Networks (1D-CNNs) and Bi-directional Long Short-term Memory networks (Bi-LSTM) serve as the foundation for the proposed model. The former extracts the local trend features and spatial correlation features, while the latter one is utilized to learn spatial-temporal connections.

While these models demonstrate the power of ensembling, they primarily focus on feature optimization for specific hydrological variables. Our work differs by addressing the structural limitations of tree-based algorithms—specifically the lack of gradient continuity—and using a physics-derived dataset to enforce strict physical consistency in a multi-stage sequential framework.

## Formulation of the problem

The transport of pollutants and dissolved oxygen (DO) in the river is modeled using a coupled one-dimensional advection–dispersion framework. The model accounts for advection, longitudinal dispersion, pollutant degradation, oxygen consumption, and atmospheric reaeration. The unsteady transport process is governed by a system of coupled one-dimensional advection–dispersion equations for the pollutant concentration $\:\mathrm{P}(x,\mathrm{t})$ (kg km^-3^) and the dissolved oxygen concentration $\:\mathrm{X}(x,\mathrm{t})$ (kg km^-3^), where t (day) denotes time. The coupled equations can be expressed as follows:

1 $$\:\frac{\partial\:P}{\partial\:t}={D}_{p}\frac{{\partial\:}^{2}P}{\partial\:{x}^{2}}-u\frac{\partial\:P}{\partial\:x}-{K}_{1}\frac{X}{X+{K}_{3}}\:P+\mu\:\:\left(1-\mathrm{exp}\left(-\lambda\:x\right)\right),$$

2 $$\:\frac{\partial\:X}{\partial\:t}={D}_{X}\frac{{\partial\:}^{2}X}{\partial\:{x}^{2}}-u\frac{\partial\:X}{\partial\:x}-{K}_{2}\frac{X}{X+{K}_{3}}P+\alpha\:\:\left(S-X\right),$$

where, $\:{D}_{p}\:\left({{km}^{2}\:day}^{-1}\right)$ is the diffusion coefficient of the pollutant concentration, $\:{D}_{X}\:\left({{km}^{2}\:day}^{-1}\right)$ is the diffusion coefficient of DO, $\:u\:\left({km\:day}^{-1}\right)$ is the flow velocity, $\:{K}_{1}\:\left({day}^{-1}\right)$ is the degradation rate coefficient for pollution at a constant temperature, $\:{K}_{2}\:\left({day}^{-1}\right)$ is the degradation rate coefficient for DO at a constant temperature, $\:{K}_{3}\left({kg\:km}^{-3}\right)$ is the half-saturated oxygen demand concentration for pollution decay, $\:\mu\:\left({kg\:km}^{-1}{day}^{-1}\right)$ is the rate of the injected pollutant discharge, $\:\lambda\:$ is spatial decay parameter of the pollutant discharge source, $\:\alpha\:\:\left({day}^{-1}\right)$ is the reaeration coefficient representing the oxygen transfer rate from the atmosphere to the water and $\:S\left({kg\:km}^{-3}\right)$ is saturation concentration of dissolved oxygen in water.

In Eq. (1), the term $\:\frac{\partial\:P}{\partial\:t}\:$represents the temporal variation of the pollutant concentration. The diffusion term $\:{D}_{p}\frac{{\partial\:}^{2}P}{\partial\:{x}^{2}}$ describes the longitudinal dispersion of pollutants caused by molecular diffusion and turbulent mixing along the river. The advection term $\:-u\frac{\partial\:P}{\partial\:x}\:$ represents the downstream transport of pollutants due to the river flow velocity. The nonlinear reaction term $\:-{K}_{1}\frac{X}{X+{K}_{3}}\:P$ models the degradation of pollutants through oxygen-dependent biochemical processes, where the degradation rate increases with dissolved oxygen availability. The source term $\:{\upmu\:}\:\left(1-\mathrm{exp}\left(-\lambda\:x\right)\right)\:$ represents the spatially distributed pollutant loading along the river, where the exponential function accounts for the gradual spatial decay of the pollutant discharge source.

Similarly, in Eq. (2), the term $\:\frac{\partial\:X}{\partial\:t}\:$ denotes the temporal variation of dissolved oxygen concentration. The diffusion term $\:{D}_{X}\frac{{\partial\:}^{2}X}{\partial\:{x}^{2}}\:$ represents the longitudinal dispersion of dissolved oxygen within the river. The advection term $\:-u\frac{\partial\:X}{\partial\:x}\:$ describes the transport of dissolved oxygen by the flowing water. The nonlinear consumption term $\:-{K}_{2}\frac{X}{X+{K}_{3}}P$ characterizes the depletion of dissolved oxygen due to biochemical pollutant degradation processes. Finally, the reaeration term $\:\alpha\:\:\left(S-X\right)\:$ represents the atmospheric oxygen transfer from air to water, which drives the dissolved oxygen concentration toward the saturation level (S).

In this study, the river is assumed to be polluted as a result of previous industrial activities. To mitigate the existing pollution, clean oxygen-rich water is released at the upstream boundary ($\:x=0$). The river initially contains dissolved pollutants before the remediation process starts. Accordingly, the initial and boundary conditions associated with Eqs. (1)–(2) describe the initial spatial distributions of pollutant concentration and dissolved oxygen along the river. At the upstream boundary, the pollutant concentration is assumed to vanish due to the injection of clean water, while a prescribed dissolved oxygen concentration is maintained. The far-field boundary conditions assume negligible concentration gradients sufficiently far downstream. The corresponding initial and boundary conditions are given by:

3 $$\:P\left(x,t\right)={P}_{0}+{P}_{1}\:\:{e}^{\frac{-x}{{K}_{4}}},\:x\ge\:0,\:t=0,$$

4 $$\:P\left(x,t\right)=0,\:\:\:\:\:\:x=0,\:t>0,$$

5 $$\:\frac{\partial\:P}{\partial\:x}=0,\:x\to\:\infty\:,\:\:\:t\ge\:0,$$

6 $$\:X\left(x,t\right)={X}_{0}{{+X}_{1}e}^{\frac{-x}{{K}_{5}}}\:,\:\:x\ge\:0,\:t=0,$$

7 $$\:X\left(x,t\right)={X}_{2},\:x=0,\:t>0,$$

8 $$\:\frac{\partial\:X}{\partial\:x}=0,\:x\to\:\infty\:,\:t\ge\:0,$$

where$\:\:{P}_{0}$ $\:(\mathrm{k}\mathrm{g}\:{km}^{-\:3}$) and $\:{X}_{0}\:\left(\mathrm{k}\mathrm{g}\:{km}^{-\:3}\right)$ are the initial concentrations for pollution and DO at $\:x\to\:\infty\:$, $\:{\mathrm{P}}_{1}\left(\mathrm{k}\mathrm{g}\:{km}^{-\:3}\right)$ and$\:\:{X}_{1}\left(\mathrm{k}\mathrm{g}\:{km}^{-\:3}\right)$ are constant. $\:{K}_{4}\left(\mathrm{k}\mathrm{m}\right)$ is the initial pollutant-decay length and $\:{K}_{5}\left(\mathrm{k}\mathrm{m}\right)$ is initial DO growth length. $\:{X}_{2}\left(\mathrm{k}\mathrm{g}\:{km}^{-\:3}\right)$ represents the dissolved oxygen concentration of the clean water released at the upstream boundary $\:x=0.$ This value is typically close to, or equal to, the saturation concentration $\:S\:$, reflecting oxygen-rich inflow used for river flushing and remediation.

### Real-world physical description for the problem

This study considers a natural river system that has been polluted as a result of previous industrial activities, leading to the presence of dissolved pollutants along the river before the implementation of any remediation measures. This pollution causes a deterioration in water quality and a reduction in dissolved oxygen levels, which adversely affect the river ecosystem and aquatic life. The problem does not aim to model an active industrial discharge; rather, it focuses on mitigating the impact of existing pollution through controlled environmental remediation strategies. To improve water quality, clean water is released into the river at the upstream location $\:x=0$. The injected water is assumed to be free of pollutants and rich in dissolved oxygen, representing a controlled river-flushing and remediation process. The clean-water release starts at $\:t=0$ and continues over time, allowing the river system to gradually recover in the downstream direction. As a result, pollutant concentrations decrease due to dilution, transport, and physical, chemical, and biological degradation processes, while dissolved oxygen levels increase due to direct oxygen supply in addition to atmospheric reaeration. Controlling the flow velocity at the clean-water injection point is a key element of the remediation strategy. The inlet velocity $\:u$ reflects the amount of clean water introduced into the river and therefore directly influences pollutant transport, dispersion, degradation rates, and dissolved oxygen dynamics. Increasing the flow velocity enhances longitudinal mixing and accelerates downstream transport, while also improving oxygen availability. However, excessive clean-water release may lead to inefficient water use without proportional environmental benefits, highlighting the need to identify an optimal flow velocity. From an ecological perspective, the effectiveness of the remediation process is evaluated based on dissolved oxygen availability, which is a critical factor for aquatic life. It is well known that fish inhabit regions where the dissolved oxygen concentration exceeds 30% of the saturation concentration. Accordingly, this threshold is adopted as a criterion for defining environmentally safe conditions along the river. Ensuring that dissolved oxygen levels remain above this limit throughout the river reach is therefore a primary objective of the clean-water release strategy. By linking the flow velocity with the coupled dynamics of pollutant concentration and dissolved oxygen, the proposed framework enables the identification of river regions capable of sustaining aquatic life. Regions that satisfy the oxygen threshold are classified as environmentally safe habitats, which support the delineation of suitable fishing zones and the selection of appropriate locations for fish farming along the river. At the same time, the model allows for the determination of the minimum amount of clean water required to achieve these environmental objectives through appropriate control of the inlet velocity. This approach helps avoid unnecessary water release and supports efficient and sustainable water resource management, while balancing ecological protection with responsible water use.

### The numerical solution

The explicit finite difference method is applied to solve Eqs. (1) and (2) associated with the initial and boundary conditions (3–8). The central difference scheme was used for $\:\frac{{\partial\:}^{2}P}{\partial\:{x}^{2}}$, $\:\:\frac{{\partial\:}^{2}X}{\partial\:{x}^{2}}$, $\:\:\frac{\partial\:P}{\partial\:x}$ and $\:\frac{\partial\:X}{\partial\:x}$. The forward difference scheme was used for $\:\frac{\partial\:P}{\partial\:t}\:$ and $\:\frac{\partial\:X}{\partial\:t}$. With these substitutions, Eqs. (1) and (2) can be written as:

9 $$\:{P}_{i,j+1}={r}_{1}{P}_{i-1,j}+{r}_{2}{P}_{i,j}+\:{r}_{3}{P}_{i+1,j}-\varDelta\:t\left({K}_{1}\frac{{X}_{i,j}}{{X}_{i,j}+{K}_{3}}\right)\:{P}_{i,j}\:\:\:+\mu\:\:\varDelta\:t\:\left(1-{e}^{-\:\lambda\:\:{x}_{i}}\right)\:,$$

10 $$\:{X}_{i,j+1}={r}_{4}{X}_{i-1,j}+{r}_{5}{X}_{i,j}+\:{r}_{6}{X}_{i+1,j}+{C}_{i,j}-\:{\Delta\:}t\left(\:{K}_{2}\:\:\frac{{X}_{i,j}}{{X}_{i,j}+{K}_{3}}\right)\:{P}_{i,j}\:+\:{\upalpha\:}\:{\Delta\:}t\:\left(S-{X}_{i,j}\right)\:\:$$

where *i* and *j* refer to the discrete step lengths $\:\varDelta\:x$ and $\:\varDelta\:t$ for the coordinate *x* and time *t*, respectively, and

$$\:{r}_{1}=\varDelta\:t\left(\frac{{D}_{\mathrm{p}}\:}{{\left({\Delta\:}x\right)}^{2}}+\:\frac{u}{2\left({\Delta\:}x\right)}\right)\:\:,\:{r}_{2}=1-\frac{2{D}_{\mathrm{p}}\:{\Delta\:}t}{\:{\left({\Delta\:}x\right)}^{2}}\:,\:{r}_{3}={\Delta\:}t\left(\frac{{D}_{\mathrm{p}}\:\:}{{\left({\Delta\:}x\right)}^{2}}-\:\frac{u\:}{2\left({\Delta\:}x\right)}\:\right),\:$$

$$\:{r}_{4}={\Delta\:}t\left(\frac{{D}_{\mathrm{X}}\:}{{\left({\Delta\:}x\right)}^{2}}+\:\frac{u\:}{2\left({\Delta\:}x\right)}\right)\:,\:{r}_{5}=1-\frac{2{D}_{\mathrm{X}}\:{\Delta\:}t}{\:{\left({\Delta\:}x\right)}^{2}}\:\:\mathrm{a}\mathrm{n}\mathrm{d}\:{r}_{6}={\Delta\:}t\left(\frac{{D}_{\mathrm{X}}\:\:}{{\left({\Delta\:}x\right)}^{2}}-\:\frac{u\:}{2\left({\Delta\:}x\right)}\:\right).\:\:$$

The initial and boundary conditions for the two Eqs. (9) and (10) can be expressed in the finite difference form as

11 $$\:{P}_{i,0}={P}_{0}+{P}_{1}{e}^{-\frac{{x}_{i}}{{K}_{4}}}\:,\:\:\:\:\:\:\:\:x\ge\:0\:,\:t=0$$

12 $$\:{P}_{0,j}=0,\:\:\:\:\:\:\:x=0\hspace{1em}t>0$$

13 $$\:{P}_{N,j}={P}_{N-1,j}\:,\:x\:\to\:\:\infty\:\:\:,\:\:t\ge\:0$$

14 $$\:{X}_{i,0}={X}_{0}+{X}_{1}{e}^{-\frac{{x}_{i}}{{K}_{5}}}\:,\:\:\:\:\:\:x\ge\:0\:,\:t=0$$

15 $$\:{X}_{0,j}={X}_{2},\:\:\:\:\:\:\:\:x=0\:t>0$$

16 $$\:{X}_{N,j}={X}_{N-1,j\:},\:\:\:\:x\:\to\:\:\infty\:,\:\:t\ge\:0\:\:\:\:\:\:\:\:\:\:\:\:\:\:\:\:\:\:$$

where$\:\:{t}_{j}=j\:{\Delta\:}t\:$ and $\:{x}_{i}=i\:{\Delta\:}x.$ $\:N=\frac{{\:\:x}_{\infty\:}}{\varDelta\:x}\:$ is the grid dimension in the $\:x$ direction and $\:{x}_{\infty\:}\:$ is the distance from $\:x=0$ in the direction of $\:x$ at which $\:\:\:\frac{\partial\:P}{\partial\:x}\to\:0\:$ and $\:\frac{\partial\:X}{\partial\:x}\to\:0$.

### Analytical solution

To validate the accuracy of the explicit finite difference scheme, we consider a simplified version of Eq. (1) in which the reaction and source terms vanish. Setting $\:{K}_{1}=0$ and $\:{\upmu\:}=0$, and choosing the initial and boundary conditions as $\:{P}_{0}=0$, the governing equation reduces to the homogeneous advection–diffusion equation:

17 $$\:\frac{\partial\:P}{\partial\:T}={D}_{p}\frac{{\partial\:}^{2}P}{\partial\:{x}^{2}}-u\frac{\partial\:P}{\partial\:x\:},$$

with the conditions

18 $$\:P\left(x,t\right)={P}_{1}\:\:{e}^{\frac{-x}{{K}_{4}}},x\ge\:0,\:t=0,$$

19 $$\:P\left(x,t\right)=0,\:x=0,\:\:\:\:t>0,$$

20 $$\:\frac{\partial\:P}{\partial\:x}=0,\:\:\:\:\:x\to\:\infty\:,\:\:\:\:t\ge\:0$$

Applying the Laplace transformation to Eq. (17) and using the inverse Laplace transformation, we obtain the analytical solution of the advection–dispersion Eq. (17), subject to the initial and boundary conditions (18)–(20), in terms of $\:x$ and $\:t$, as follows:

21 $$\:P\left(x,t\right)={P}_{1}\left(\begin{array}{c}{\mathrm{e}}^{\left(\frac{{D}_{p}\:t}{{{K}_{4}}^{2}}+\frac{t\:u-x}{{K}_{4}}\right)}-\frac{1}{2}{\mathrm{e}}^{\left(\frac{{D}_{p}\:t}{{{K}_{4}}^{2}}+\frac{tu-x}{{K}_{4}}\right)}erfc\left[\frac{x-ut}{2\sqrt{{D}_{p}\:t}}-\frac{\sqrt{{D}_{p}\:t}}{{K}_{4}}\right]\\\:-\frac{1}{2}{\mathrm{e}}^{\left(\frac{{D}_{p}\:t}{{{K}_{4}}^{2}}+\frac{tu+x}{{K}_{4}}+\frac{ux}{{D}_{p}}\right)}erfc\left[\frac{x+ut}{2\sqrt{{D}_{p}\:t}}+\frac{\sqrt{{D}_{p}\:t}}{{K}_{4}}\right]\:\end{array}\right)$$

where erfc is the complementary error function. We have confirmed that Eq. (21) satisfies Eqs. (17–20).

## Materials and methods

**Fig. 1:**
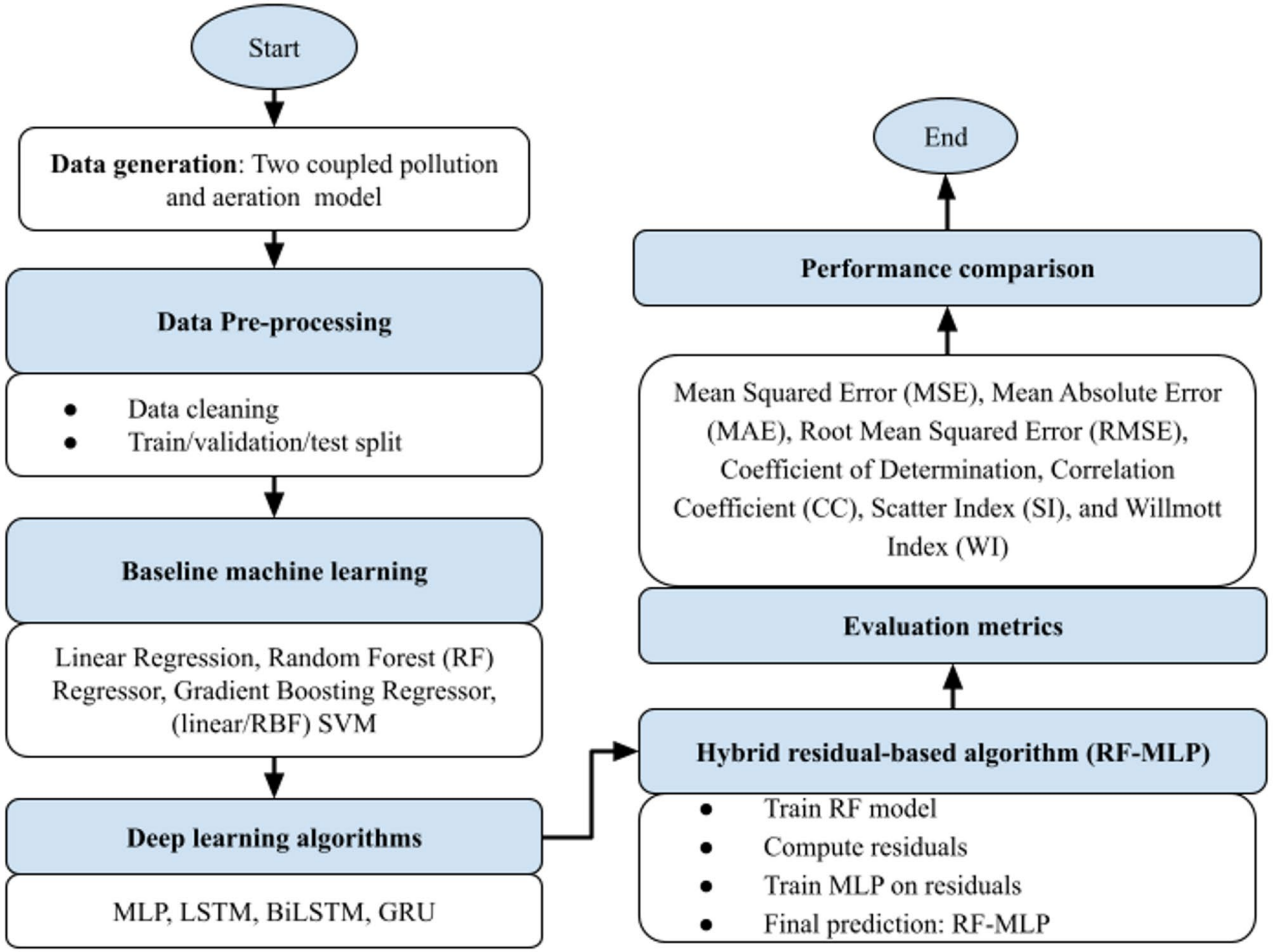
Methodology workflow.

This research adopts a systematic machine learning and deep learning framework for predicting pollutant concentration dynamics in a river system. The core of the methodology involves leveraging physically based simulation data to train and validate hybrid AI algorithm. As depicted in the methodology workflow (Fig. 1), the study follows a rigorous, multi-phase progression. The data management lifecycle—encompassing collection, pre-processing, artifact filtering, and exploratory analysis—is detailed in Sect.  4.1, while the specific partitioning of datasets for training, validation, and testing is discussed in Sect.  4.2. The benchmark baseline models used for comparison are introduced in Sect.  4.3. The architecture and logic of the proposed hybrid RF-MLP algorithm, alongside its algorithmic configuration and implementation details, are elaborated in Sect.  5. Performance benchmarking and the selection of evaluation metrics are established in Sect.  6, leading to the comprehensive result analysis and trade-off discussion provided in Sect.  7.

### Physics-based data collection, pre-processing, and analysis

The dataset used to train and evaluate the AI algorithms was derived from the two coupled pollution and aeration equations. Thus, the proposed hybrid algorithm adheres to the physical consistency of the ADE process. The dataset contains the pollution concentration $\:P\left(x,t\right)$ and dissolved oxygen concentration $\:X\left(x,t\right)$ at 50,000 specific spatial positions ($\:x\:$) across various distinct speeds ($\:u$). Each observation represents a unique spatial point ranging from $\:x=0.0\:$ to $\:x=4.0\:$, with corresponding levels for speeds $\:u\in\:\left\{\mathrm{1,6},11\right\}$. This multi-output structure allows the models to learn the spatial distribution of the pollution plume and dissolved oxygen recovery under varying hydrodynamic regimes. The primary input feature for the predictive algorithms is the spatial position ($\:x\:$), while the target variables are the concentration values $\:P\:$ and $\:X\:$ across the specified speeds. Prior to model training, comprehensive data quality assessment and pre-processing steps were implemented to ensure data integrity and optimize algorithmic performance:

1. **Integrity Check**: The dataset dimensions and data types were inspected to confirm consistency across the 50,000 observations.
2. **Handling Missing Values**: A robust pre-processing pipeline was established using a *SimpleImputer* with a “mean” strategy to address any potential numerical instabilities, although the analytical source provides a continuous dataset.
3. **Artifact Filtering**: To ensure physical plausibility, any rows containing negative concentration values—which may arise as numerical artifacts during the calculation process—were excluded. The final cleaned dataset maintained a dimension of 50,000 observations with the input feature $\:x\:$ and target outputs for $\:P\:$ and $\:X\:$ at three different flow velocities. (One input feature $\:x\:$ and six target outputs).
4. **Feature and Target Separation**: The spatial variable $\:x\:$ was isolated as the independent feature, while the concentration values $\:P\:$ and $\:X\:$ for each speed were defined as the dependent multi-output target vector.
5. **Feature Scaling**: To mitigate the impact of varying scales and accelerate the convergence of gradient-based algorithms, the data were normalized using *StandardScalar*. Thus, transforming the spatial features to have a zero mean and unit variance.

**Fig. 2:**
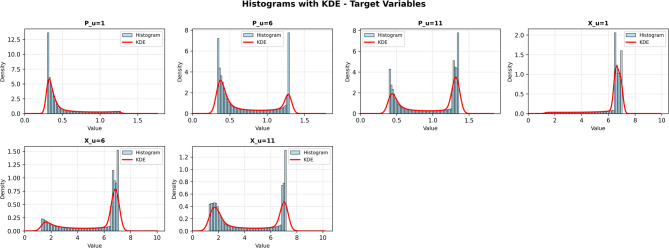
Visualizing histograms to inspect data distributions of pollutant concentrations ($\:P$) and dissolved oxygen ($\:X$) for each speed ($\:u$).

Exploratory data analysis (EDA) was performed to understand the structural and statistical characteristics of the concentrations across different hydrodynamic conditions. The results reveal strong spatial and hydrodynamic dependencies, which are critical for characterizing the non-linear transport processes captured by the ADE. The Histograms (Fig. 2) illustrate the frequency distribution of concentration values for each speed$\:\:\:u\in\:\left\{\mathrm{1,6},11\right\}$. For pollution concentration ($\:P\:$), the distributions are bimodal distributions, reflecting the distinct phases of plume rise and stabilization across the spatial domain. This pattern indicates that at most spatial positions ($\:x\:$), the concentration oscillates between initial low-level states and stabilized peak regions. Conversely, the dissolved oxygen ($\:X\:$) profiles demonstrate a depletion and sag pattern, where concentrations decrease significantly as the water moves farther from the upstream boundary, indicating a strong negative correlation with spatial position $\:x\:$.

**Fig. 3:**
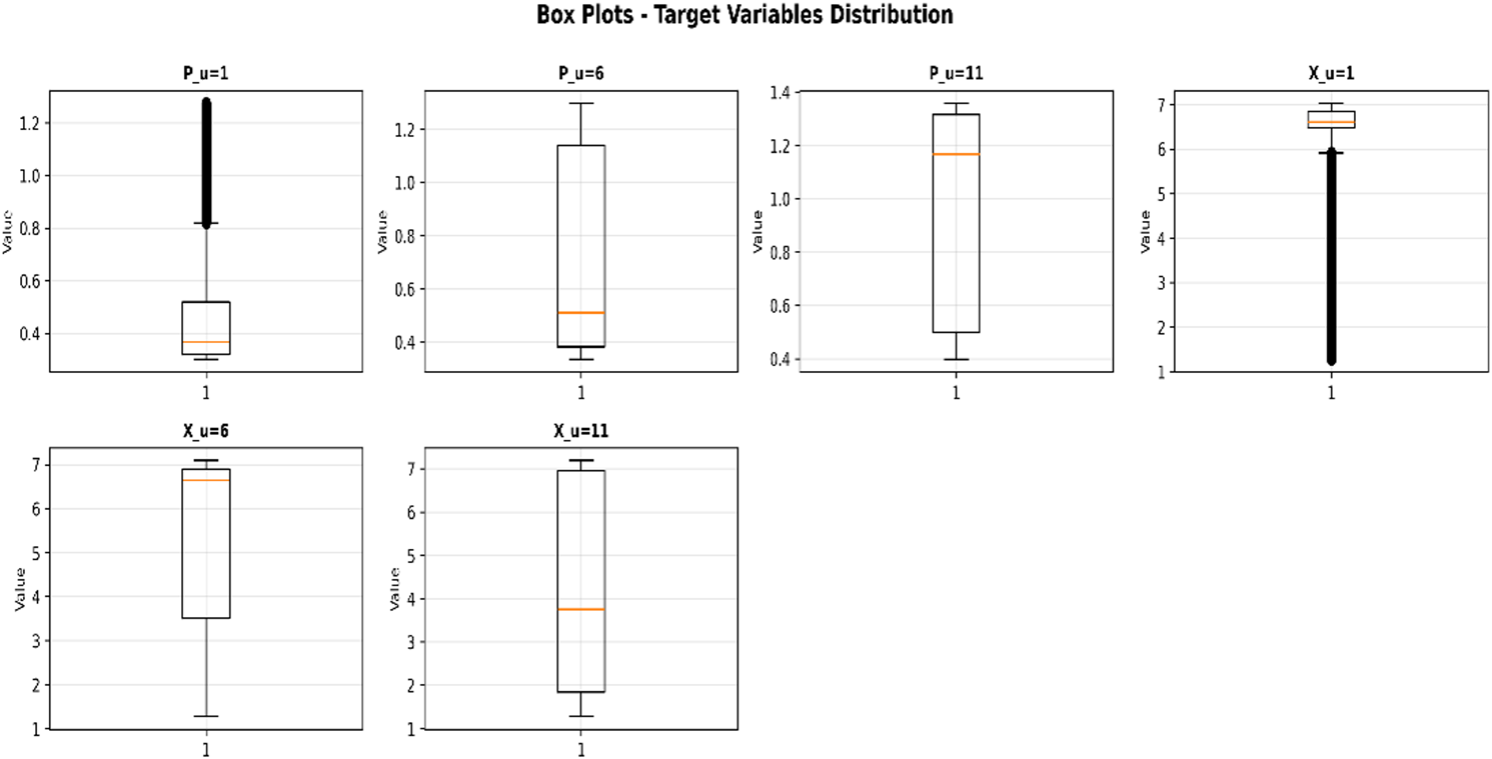
Box plots to detect potential outliers and assess variability.

**Fig. 4:**
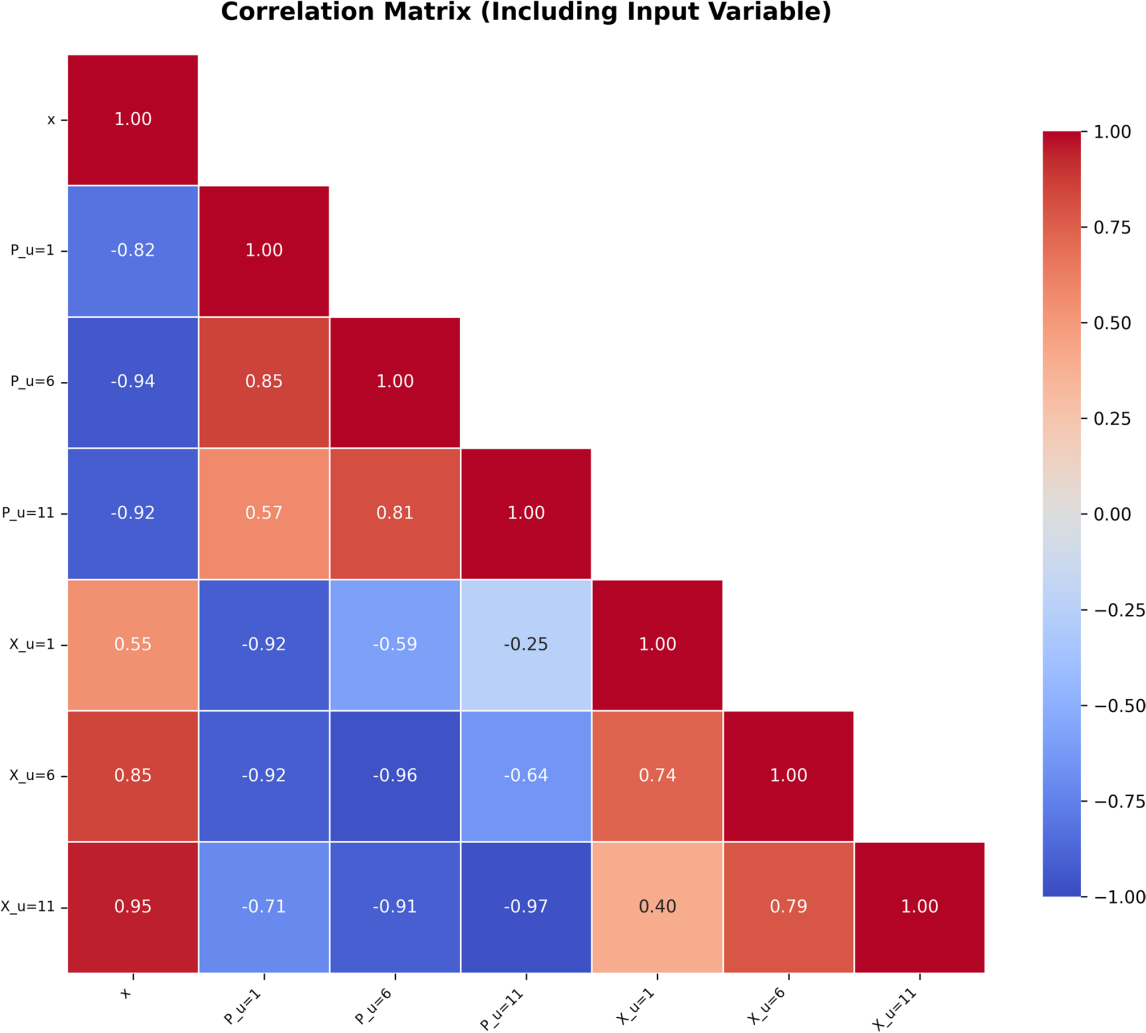
Correlation matrix to analyze the relationships between pollutant and dissolved oxygen concentrations at different speeds.

The Box Plots (Fig. 3) and Correlation Matrix (Fig. 4) further highlight the internal structure of the multi-output vector. The box plots reveal that for pollution concentration $\:P\:$, the majority of data points fall within a narrow interquartile range (IQR). Notably, the data points plotted beyond the whiskers, particularly visible for $\:{P}_{u=11}$, are not indicative of measurement noise or data errors. Since the input data is received from the solution of the coupled equations, these “statistical (not real) outliers” are physically significant, as they represent the high-concentration peaks and map the core of the pollutant plume moving through the spatial domain. In contrast, the dissolved oxygen $\:X\:$ shows a much broader variability, reflecting its complex recovery curve across the spatial domain. The correlation matrix confirms an extremely high inter-variable correlation ($\:\rho\:\approx\:0.99\:$) between the concentrations at different speeds. Interestingly, spatial position $\:x\:$shows a strong negative correlation with $\:{P}_{u=6}$ and a strong positive correlation with $\:{X}_{u=11}$, quantifying the physical phenomena of pollutant attenuation and oxygen re-aeration as water moves downstream.

The descriptive statistics for the input feature and target variables are summarized in Table 1. The spatial coordinate $\:x\:$ is uniformly distributed across the domain [0, 4.0]. For the target concentration$\:\:\:P$, both the median and the standard deviation increase monotonically with speed. For instance, at $\:u=1$, the median concentration is approximately 0.41 with a standard deviation of 0.14. By $\:u=11$, the median rises to 0.43 and the standard deviation to approximately 0.25. The 75th percentile also shifts from 0.55 (at $\:\:u=1$) to 0.60 (at $\:u=11$), confirming that higher flow velocities result in a broader spatial distribution of concentration levels across the extended spatial domain. These statistics confirm the complexity and nonlinearity of the modeled coupled system.

**Table 1:** Descriptive statistical analysis for the pollutant dataset.

Variable	Mean	Std dev	Min	Q1	Median	Q3	Max
X	2	1.15	0	1	2	3	4
Pu = 1	0.43	0.14	0	0.35	0.41	0.55	0.7
Pu = 6	0.44	0.16	0	0.35	0.42	0.58	0.75
Pu = 11	0.38	0.25	0	0.08	0.43	0.6	0.78
Xu = 1	2.31	0.48	2.2	2.2	2.21	2.22	6.5
Xu = 6	2.85	1.12	2.21	2.22	2.24	3.15	6.5
Xu = 11	4.15	1.85	2.22	2.25	4.21	6.15	6.5

### Training, validation, and testing strategy

To prevent overfitting and ensure robust evaluation, the dataset was partitioned into three distinct subsets using a two-phase splitting strategy to ensure both robust hyperparameter tuning and unbiased performance evaluation:

- **Initial Split**: An 80/20 split was first applied to separate the Testing Set (10,000 samples) from the initial training pool.
- **Secondary Split**: The remaining training pool was further divided using an 80/20 ratio to create a Validation Set for hyperparameter tuning and early stopping, and a final Training Set.

The resulting final distribution consists of approximately 64% training data (32,000 samples), 16% validation data (8,000 samples), and 20% testing data (10,000 samples). A fixed random state was maintained throughout to ensure the reproducibility of the experimental results.

### Baseline and comparison models

Before detailing the proposed hybrid algorithm, several standard machine and deep learning algorithms were implemented to establish performance benchmarks.

1. **Linear Regression**: Linear Regression is one of the simplest supervised learning models and assumes a linear relationship between the independent variables and the target variable. Despite its simplicity, it is frequently used as a baseline model for evaluating the performance of more complex machine learning algorithms.
2. **Support Vector Machine (linear/RBF SVM)**: Support Vector Machines aim to find an optimal separating hyperplane between data points in the feature space. For nonlinear classification problems, kernel functions such as the Radial Basis Function (RBF) kernel are used to project data into a higher-dimensional space where linear separation becomes feasible. Linear SVM is commonly applied when data are linearly separable or when dealing with high-dimensional feature spaces.
3. **Random Forest (RF)**: Random Forest (RF) is an ensemble algorithm that constructs multiple decision trees and aggregates their outputs to produce a more accurate and stable prediction^66^. Each tree is trained using bootstrap sampling, and at each split a random subset of features is considered. This randomness reduces correlation among trees, lowering variance and improving generalization. In classification, predictions are made via majority voting, while regression uses averaging of tree outputs. RF is widely used due to robustness to noise, ability to model nonlinear patterns, and strong performance with high‑dimensional data.
4. **Multi-Layer Perceptron (MLP)**: A Multilayer Perceptron (MLP) is a feed‑forward neural network composed of an input layer, one or more hidden layers, and an output layer. Each neuron performs a weighted sum followed by a nonlinear activation, enabling the network to model complex relationships^67^. MLPs are trained using backpropagation, and their flexibility in architecture and activation functions makes them suitable for nonlinear prediction tasks. MLPs also have strong approximation capabilities.
5. **Gradient Boosting**: Gradient Boosting is an ensemble learning technique that builds a sequence of weak learners, typically decision trees, where each subsequent model is trained to reduce the residual errors of the previous one. It is the most recent algorithm employed in competitions. It uses an additive model that allows for the optimization of a differentiable loss function. The optimization process minimizes a loss function using gradient descent, leading to strong predictive performance, especially in nonlinear problems^68^.
6. **Recurrent Neural Networks (GRU**,** LSTM**,** and BiLSTM)**: Recurrent Neural Networks (RNNs) are designed to model sequential and time-dependent data by maintaining internal memory of past inputs. Long Short-Term Memory (LSTM) networks address the vanishing gradient problem through gating mechanisms that regulate information flow^69^. Gated Recurrent Units (GRU) provides a simplified alternative to LSTM, reducing the number of parameters while maintaining competitive learning performance. Bidirectional LSTM (BiLSTM) processes sequences in both forward and backward directions, enabling the model to capture past and future temporal dependencies more effectively^70^.

## Computational framework and models architecture

### The proposed heterogeneous boosting physics-guided hybrid (RF-MLP) algorithm

The proposed RF-MLP algorithm utilizes a heterogeneous boosting architecture that differs fundamentally from standard ensemble techniques in its underlying philosophy and structural execution. While standard stacking typically employs multiple base learners whose outputs are aggregated by a meta-learner, and standard boosting (e.g., AdaBoost or Gradient Boosting) relies on a sequence of homogeneous weak learners, our approach follows a three-stage sequential logic:

**Stage 1 (Initial Mapping):** The Random Forest base learner generates an initial prediction of the concentration values. This stage captures the primary non-linear trend of the advection-dispersion process. The choice of Random Forest (RF) as the primary layer is motivated by its inherent stability in handling high-dimensional environmental data and its ability to identify hierarchical “regimes” in spatial data. In fluvial transport, the pollutant profile is split into distinct regions (e.g., arrival, peak, and decay phases). RF’s decision-tree structure is uniquely suited to partition the feature space into these segments without requiring extensive manual feature engineering. RF establishes the global magnitude “backbone” of the plume, effectively reducing variance and identifying non-linear patterns that baseline linear models fail to capture.

**Stage 2 (Residual Refinement Layer):** While RF provides a robust magnitude estimate, its output is piecewise-constant, leading to “step-like” artifacts that violate the continuous physical gradients of diffusion. Thus, an MLP layer, unlike simple “correctors” that merely add a bias term, is then trained to predict the residuals (errors) of the RF. As a universal function approximator, the MLP excels at learning continuous relationships. Because the MLP’s target is the “unaccounted variance” rather than the raw data, it can focus specifically on refining the boundaries where the RF’s decision trees are less precise, restoring the smooth exponential curvature and high-frequency transitions required for physical fidelity to the coupled equations.

**Stage 3 (Synthesis):** The final output is the sum of the RF prediction and the MLP-corrected residual. The hybrid framework concludes after the MLP stage because additional layers introduce unnecessary complexity with negligible performance gains. As will be shown in the “results section”, the Hybrid RF + MLP (Residual) Corrected model already achieves a Willmott’s Index of 0.999999998635489. Further layers would provide the risk of overfitting to the training data and increase computational latency, which would undermine the model’s utility for real-time environmental monitoring.

This architecture ensures that the model does not merely perform ‘function approximation’ but internalizes the governing physics, resulting in superior physical fidelity compared to standalone or traditionally stacked models.

### Algorithmic implementation and hyperparameter selection

The hyperparameters for all machine learning and deep learning algorithms employed in this study were meticulously defined to ensure reliable performance and a fair comparison across different modeling algorithms. The final configurations for each model/algorithm are summarized in Table 2. These parameters were selected based on empirical experimentation and validation performance, aiming to achieve an optimal balance between predictive accuracy, generalization capability, and computational efficiency. For the Random Forest (RF) model, a total of 100 trees were utilized to construct the ensemble, providing stable predictions while minimizing model variance. A fixed random state of 42 was applied to ensure the reproducibility of all experimental results. The Multi-Layer Perceptron (MLP) was configured with three hidden layers consisting of 100, 100, and 50 neurons, respectively, enabling the network to approximate complex nonlinear mappings effectively. The Rectified Linear Unit (ReLU) activation function was used in the hidden layers to facilitate gradient flow, while the Adam optimizer was employed to manage the learning rate during training. For the sequential deep learning models, LSTM, BiLSTM, and GRU architectures were implemented with 50 hidden units. The tanh activation function was utilized within the recurrent layers to handle gated state transitions, and the models were trained using the Adam optimizer with a Mean Squared Error (MSE) loss function. To prevent overfitting and ensure effective generalization to the test set, we employed an Early Stopping callback with a patience of 15 epochs, monitoring the validation loss to restore the best performing weights.

**Table 2:** Hyperparameter configuration of the employed machine learning, hybrid, and deep learning algorithms.

Model/algorithm	Hyperparameter	Value/setting
Linear regression (LR)	Regularization	None (ordinary least squares)
	Intercept	fit_intercept=True
Gradient boosting (GB)	Number of estimators	100
	Learning rate	0.1
	Maximum depth	3
Random forest (RF)	Number of trees ($n\_estimators$)	100
	Random state	42
Multi-layer perceptron (MLP)	Hidden layer structure	(100, 100, 50)
	Activation function	ReLU
	Optimizer	Adam
	Maximum iterations	2000
	Early stopping	Enabled (Patience = 15)
Hybrid RF–MLP (residual)	Base learner	Random Forest (*n* = 100)
	Residual learner	MLP (as configured above)
	Strategy	Residual learning
LSTM/BiLSTM/GRU	Hidden units	50
	Activation function	Tanh
	Optimizer / Loss	Adam / MSE

The implementation was executed in a Python environment on the Google Colab platform. Traditional machine learning algorithms were implemented using the Scikit-learn library, with models such as Gradient Boosting and SVM wrapped in a MultiOutputRegressor to handle the simultaneous prediction of concentrations across multiple spatial locations. The deep learning architectures were developed using the TensorFlow/Keras framework, utilizing the Sequential API for model construction.

## Evaluation metrics

Prior to presenting the obtained results, we will outline the various measures utilized to evaluate the performance of the proposed algorithm in comparison to state-of-the-art algorithms. The evaluation of predictive models for river pollution necessitates a multifaceted statistical approach, as no single metric can fully characterize the nuances of model fit, variance explanation, and potential for generalization^71^. In all subsequent equations, $\:n\:$ denotes the number of samples, $\:{y}_{i}$ represents the observed analytical concentration, $\:\widehat{{y}_{i}}$ is the predicted concentration, and $\:\overline{y}$ is the mean of the observed values.

### Statistical performance metrics

**Absolute Error Metrics** provide an assessment of the magnitude of error (deviation between predicted and observed pollution concentrations) in the same physical units as the pollutant concentration. Thus, absolute error metrics are crucial for understanding the model’s reliability in practical units. For regression, we used the following metrics:

- **Mean Absolute Error (MAE)**: Measures the average magnitude of errors without considering direction. It provides a robust ground-truth of expected error in the same units as the target variable.

$$\:\mathrm{MAE}=\frac{1}{n}{\sum\:}_{i=1}^{n}\left|{y}_{i}-\widehat{{y}_{i}}\right|$$

- **Mean Squared Error (MSE)**: Penalizes larger errors quadratically. A lower MSE in the Hybrid model indicates a significant reduction in “outlier” predictions compared to traditional methods.

$$\:\mathrm{MSE}=\frac{1}{n}{\sum\:}_{i=1}^{n}{\left({y}_{i}-\widehat{{y}_{i}}\right)}^{2}$$

- **Root Mean Squared Error (RMSE)**: The square root of MSE, providing an error metric in the original units while remaining sensitive to large deviations.

$$\:\mathrm{RMSE}=\sqrt{\frac{1}{n}{\sum\:}_{i=1}^{n}{\left({y}_{i}-\widehat{{y}_{i}}\right)}^{2}}$$

MSE and RMSE penalize larger errors more heavily than MAE, making it a rigorous indicator of model stability in the presence of pollution spikes or outliers.

***Correlation and Agreement*** metrics are dimensionless indices that evaluate how well the model captures the trend and variance of the pollutant plume:

- **Coefficient of Determination (**$\:{R}^{2}$**)**: Quantifies the proportion of the variance in the target variable that is predictable from the input features.

$$\:{R}^{2}=1-\frac{{\sum\:}_{i=1}^{n}{\left({y}_{i}-\widehat{{y}_{i}}\right)}^{2}}{{\sum\:}_{i=1}^{n}{\left({y}_{i}-\overline{y}\right)}^{2}}$$

- **Correlation Coefficient (CC)**: Measures the strength and direction of the linear relationship between the analytical and predicted values.

$$\:\mathrm{CC}=\frac{{\sum\:}_{i=1}^{n}\left({y}_{i}-\overline{y}\right)\left(\widehat{{y}_{i}}-\overline{\widehat{y}}\right)}{\sqrt{{\sum\:}_{i=1}^{n}{\left({y}_{i}-\overline{y}\right)}^{2{\sum\:}_{i=1}^{n}{\left(\widehat{{y}_{i}}-\overline{\widehat{y}}\right)}^{2}}}}$$

- **Willmott’s Index of Agreement (WI)**: A standardized measure of the degree of model prediction error by comparing the accuracy of a model to a reference model based on the mean of the observed data. It varies between 0 and 1, where 1 indicates a perfect match:

$$\:\mathrm{WI}=1-\frac{{\sum\:}_{i=1}^{n}{\left({y}_{i}-\widehat{{y}_{i}}\right)}^{2}}{{\sum\:}_{i=1}^{n}{\left(\left|\widehat{{y}_{i}}-\overline{y}\right|+\left|{y}_{i}-\overline{y}\right|\right)}^{2}}$$

- **Scattered Index (SI)** is a normalized measure of the RMSE, used to compare the precision of the models relative to the mean of the observations to provide a measure of relative dispersion.

$$\:\mathrm{SI}=\frac{\mathrm{RMSE}}{\overline{y}}$$

### Computational efficiency

**Training and prediction time**: Given the requirements for potential real-time water quality monitoring or high frequency forecasting, the total CPU/GPU time required for model convergence (Training Time) and the latency for a single forward pass (Prediction Time) were recorded. These metrics were calculated for both validation and testing phases to ensure robustness and mitigate the risks of overfitting, which is a common concern in high-dimensional machine learning applications.

## Result analysis and discussion

### Statistical performance analysis

Table 3 summarizes the statistical comparative performance across all evaluated algorithms during the test phase.

**Table 3:** Statistical metrics comparison between different ML models during test phase.

Algorithm	Test MSE	Test MAE	Test RMSE	Test *R*^2^	Test CC	Test SI	Test WI
Hybrid RF + MLP (Residual)	1.68E-08	4.15E-05	0.0001296643597	0.9999999692	0.9999999973	7.33E-05	0.9999999986
Random Forest	1.86E-08	4.45E-05	0.0001364927691	0.9999999654	0.9999999971	7.71E-05	0.9999999985
BiLSTM	7.27E-06	0.001033064674	0.002696170787	0.9999805052	0.9999988415	0.001523378756	0.9999994099
LSTM	9.15E-06	0.001435921404	0.003025226684	0.9999775467	0.9999985241	0.001709300496	0.9999992573
GRU	9.70E-06	0.001450385887	0.003114295465	0.9999735048	0.9999984425	0.001759625753	0.9999992128
Gradient Boosting	1.44E-05	0.001756304928	0.003800628095	0.9999408265	0.9999976555	0.002147414447	0.9999988277
MLP	0.0001220924992	0.007370591217	0.01104954747	0.9998303694	0.9999847744	0.006243167515	0.999990065
SVM (RBF Kernel)	0.00550341716	0.05071562836	0.07418501978	0.9951937779	0.9991070513	0.04191569899	0.9995528646
Linear Regression	0.3807238371	0.4160820084	0.6170282304	0.3905649953	0.9361820953	0.3486306218	0.9658745523
Linear SVM	0.5464185661	0.3756462115	0.7392013028	0.1467051906	0.9091621678	0.4176603227	0.9474468911

The results demonstrate that the proposed hybrid (RF-MLP) model consistently outperforms all other architectures, achieving near-perfect metrics with a Test RMSE of 0.00012966 and an $\:{R}^{2}$ of 0.9999999692. This high level of precision is indicative of the model’s ability to not only learn the primary structural patterns of the data via the RF base but also to refine these predictions by capturing the residual errors through an MLP corrector. While standalone ensemble models achieve high accuracy, the Hybrid model, by adding the MLP corrector, further reduces the error, representing a consistent refinement in predictive precision. This suggests that the residual term contains significant deterministic patterns—likely non-linear spatial dependencies that the tree-based splits of the RF could not fully resolve—which the MLP is adept at identifying. This dual-stage modeling respects the structural strengths of both algorithms: the RF provides a stable, low-variance baseline, while the MLP provides a high-capacity non-linear refinement. Deep learning algorithms like LSTM and GRU are frequently proposed as solutions for datasets with complex dependencies. Indeed, the results show that LSTM and GRU perform exceptionally well ($\:{R}^{2}\approx\:0.999$). However, they do not surpass the hybrid RF-MLP in this specific dataset spanning the expanded spatial domain [0, 4.0]. The progression of error increases as the analysis moves toward these deep learning architectures (Test RMSE = 0.003114 for GRU, and 0.003025 for LSTM), suggesting that while these models are adept at capturing dependencies, they may require more extensive hyperparameter tuning or larger datasets to match the precision of refined ensemble hybrids in this specific context. A possible explanation is that the hybrid model leverages the strengths of ensemble learning, which is often more data-efficient than the deep recurrent structures of LSTMs when the dataset size is finite. Furthermore, the residual correction strategy allows the model to explicitly target the ‘hardest’ part of the prediction task—the errors—rather than attempting to learn the entire signal at once. In contrast to the hybrid framework, linear models—specifically Linear Regression and Linear SVM—demonstrate significantly higher error rates and lower variance explanation, underscoring the inadequacy of linear approximations for environmental spatial snapshots that are inherently governed by non-linear physical interactions. A CC and $\:{R}^{2}$ of nearly 1.0, as seen in the top-performing models, indicate that these architectures have achieved near-perfect mapping of the input-output relationships. Because the training, validation, and testing partitions are synthetically generated from a deterministic, noise-free physical model (specifically, the coupled advection-dispersion-aeration equations), the machine learning algorithms are emulating a mathematically closed, continuous system rather than fitting stochastic, observationally noisy real-world field measurements. Consequently, the near-unity values of $\:{R}^{2}$ and CC reflect the high-capacity estimators’ absolute precision in emulating the idealized, noise-free physics of mass transport, rather than an expectation of identical statistical performance under unmodeled real-world environmental turbulence and sensor-level measurement errors. While CC and $\:{R}^{2}$ are high for most of the compared models, the $\:{R}^{2}$ degrades for the linear models. The Willmott Index (WI), or Index of Agreement, provides additional validation of model skill. For the top models—including Hybrid, RF, GRU, MLP, LSTM, BiLSTM, and GB—the WI is nearly 1.0, reflecting ‘exceptional accuracy’ and ‘strong agreement’ with spatially distributed pollution patterns (e.g., Hybrid WI = 0.9999999986). Even for the poorly performing linear algorithms, the agreement is noticeably lower, suggesting that they fail significantly in both absolute precision and capturing general directional trends. The compared models’ performance was evaluated based on the Scatter Index (SI) criteria, where an SI below 0.1 signifies ‘excellent’ model performance. Applying these thresholds, the Proposed Hybrid (RF-MLP), RF, GRU, LSTM, MLP, BiLSTM, and GB consistently achieved ‘excellent’ ratings with SI values significantly below the 0.1 threshold (e.g., Hybrid SI = 0.00007326). In stark contrast, the linear models yielded significantly higher SI values, placing them in lower performance categories. This disparity validates that for river pollution forecasting, the non-linear mapping capabilities of hybrid algorithms are an operational necessity for achieving reliable, high-fidelity concentration profiles.

### Computational complexity and trade-off analysis

In the context of real-time monitoring and autonomous early-warning systems, predictive fidelity must be strategically balanced against computational overhead. The deployment of pollution models in IoT-integrated sensor networks necessitates architectures that can be trained and executed within strict temporal windows, particularly as the sampling frequency of river data increases. The training and inference latencies recorded for the evaluated models reveal critical trade-offs in algorithmic efficiency as shown in Table 4.

**Table 4:** Training and prediction times comparisons between different models.

Algorithm	Train time (s)	Pred time (s)
Linear regression	0.0282	0.00207
Linear SVM	0.713	0.00403
MLP	5.51	0.1492812634
Gradient boosting	11.3	0.3772344589
Random forest	5.83	2.118192434
Hybrid RF + MLP (residual)	22.6	2.127525806
SVM (RBF kernel)	36.35931778	2.403913498
GRU	453.186209	2.737650394
LSTM	426.4549406	2.770416021
BiLSTM	552.2100234	2.919160843

#### Training latency and computational bottlenecks

The training duration serves as a proxy for the offline computational cost of model development. The deep recurrent architectures (LSTM, GRU, BiLSTM) exhibited the highest computational burden. Specifically, the GRU and BiLSTM required 453.19 s and 552.21 s, respectively, to reach convergence—representing an approximately 94.7-fold increase in training time for the BiLSTM compared to the standalone Random Forest model (5.83 s). This latency is a structural hallmark of recurrent networks, where the sequential processing of steps and the optimization of multi-gated weight matrices (input, forget, and output gates) impose a high cost on backpropagation through time (BPTT). While these models are highly effective at capturing dependencies, their training complexity poses a significant bottleneck for systems requiring frequent online retraining or deployment on edge devices with limited memory and processing power. The Proposed Hybrid RF-MLP algorithm occupies a computational “sweet spot,” with a total training time of 22.6 s. Given that this model achieved an order-of-magnitude reduction in RMSE compared to its deep learning peers (e.g., GRU at 0.003114 vs. Hybrid at 0.00012966), this represents an exceptionally high performance-per-second ratio. The marginal 16.75 s increase in training time over the standalone RF (5.83 s) is a justifiable engineering trade-off. It indicates that the MLP residual corrector, while significantly enhancing precision, remains a lightweight architectural addition compared to the massive parameter space of the deep BiLSTM.

#### Inference feasibility in real-time warning systems

For operational water quality management, inference latency (prediction time) can be more critical than training time. Once deployed, a model must ingest real-time spatial data and generate forecasts near-instantaneously to facilitate rapid emergency intervention. The MLP model demonstrated the highest inference efficiency with a prediction time of 0.1493 s for the entire test set. This speed is attributed to the feed-forward nature of the network, which, post-training, simplifies to a series of optimized matrix multiplications. In contrast, the Hybrid RF-MLP and standard RF models showed higher latencies (2.1275 s and 2.1182 s, respectively). This is due to the computational requirement of traversing a high-density forest of decision trees during the ensemble aggregation phase. However, even at approximately two to three seconds, these inference speeds are more than sufficient for sub-hourly or even minute-by-minute river monitoring. The most notable delays in this updated analysis occurred with the deep recurrent architectures, specifically the BiLSTM (2.9192 s) and LSTM (2.7704 s). For these models, the sequential processing logic and multi-gated weight matrices impose higher computational overhead compared to the proposed hybrid framework.

### Statistical stability and reliability of predictions

Table 5 summarizes the statistical comparative performance across all evaluated algorithms during validation phase.

**Table 5:** Statistical metrics between different ML models during validation phase.

Algorithm	Val MSE	Val MAE	Val RMSE	Val *R*^2^	Val CC	Val SI	Val WI
Hybrid RF + MLP (residual)	1.55E-08	3.99E-05	0.0001244237305	0.9999999714	0.9999999975	7.08E-05	0.9999999987
Random forest	1.72E-08	4.28E-05	0.0001311408103	0.9999999679	0.9999999973	7.46E-05	0.9999999986
BiLSTM	7.42E-06	0.001009292634	0.002724738895	0.9999791775	0.9999987849	0.001550693857	0.9999993828
LSTM	9.20E-06	0.001405185395	0.003033490016	0.9999763591	0.9999984804	0.001726409214	0.9999992352
GRU	9.74E-06	0.001423939619	0.003120991303	0.9999724296	0.9999983954	0.00177620764	0.9999991903
Gradient boosting	1.36E-05	0.001715599196	0.003683558934	0.9999421061	0.9999977444	0.002096374159	0.9999988722
MLP	0.0001230371729	0.007423739119	0.01109221226	0.9998260799	0.9999842219	0.006312760995	0.999989746
SVM (RBF Kernel)	0.00548118709	0.05066675407	0.07403503961	0.995089969	0.9990889253	0.04213456245	0.9995438625
Linear regression	0.3759427651	0.41338588	0.613141717	0.3885379831	0.9354180193	0.3489490666	0.9656391044
Linear SVM	0.5298192066	0.3702752619	0.7278868089	0.1544139073	0.9094579586	0.4142523914	0.9480925067

A deeper examination of the validation vs. testing metrics provides critical insights into model generalization and the potential for overfitting. In this study, the performance of the Hybrid, RF, and deep learning models is remarkably consistent between the two phases. For example, the Hybrid RF-MLP algorithm maintains a near-identical error profile across both partitions, with a Validation RMSE of 0.00012442 and a Test RMSE of 0.00012966. Consistently low error rates across both datasets indicate a highly stable architecture with high generalization skills. This stability is often attributed to the ensemble nature of the Random Forest base, which uses bagging to reduce variance. By averaging the results of many uncorrelated decision trees, the model effectively filters out the random fluctuations present in individual sampling points. Similarly, the standalone RF model displays high stability with a Validation RMSE of 0.00013114 and a Test RMSE of 0.00013649, confirming that the high predictive precision is not a result of overfitting to the training or validation data, but rather a reflection of the models’ ability to capture the underlying physical dynamics of the coupled pollution-aeration equations.

### Physical based interpretation against AI prediction results

#### AI prediction results

**Fig. 5:**
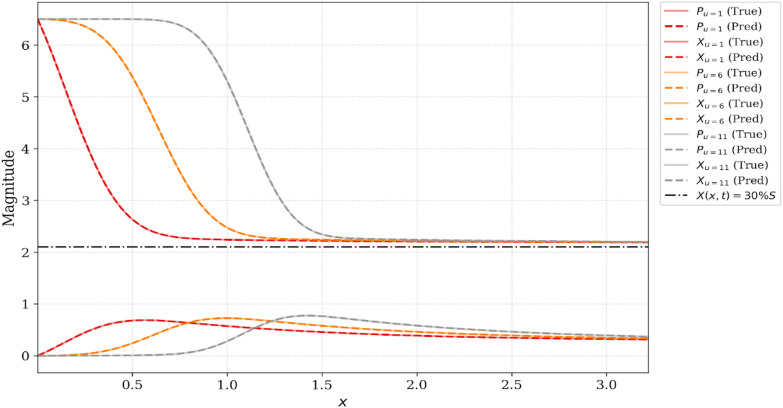
Predicted hybrid AI algorithm for the coupled water pollution and aeration concentrations vs. analytical solver actual data.

The hybrid algorithm accurately captured the distinct spatial regimes of the plume, including the near-source peak, the dispersion zone, and the downstream depletion and sag phase. By refining the RF residuals, the MLP successfully eliminated step-like artifacts, resulting in smooth pollutant concentration curves and dissolved oxygen concentration curves that are fully consistent with the numerical results obtained using the explicit finite difference method as illustrated in Fig. 5 for the spatial domain$\:\:0\:\le\:x\:\le\:4\:$. The spatial distributions of pollutant concentration $\:P\left(x,t\right)$ and dissolved oxygen concentration $\:X\left(x,t\right)$ were evaluated at flow velocities $\:u\:=\:1,\:6\:$ and $\:11\:$ along the river.

Beyond this specific case, the results highlight the broader potential of artificial intelligence to address coupled transport problems of increasing complexity. In realistic environmental systems, pollutant dynamics are often influenced by nonlinear interactions, spatial heterogeneity, and incomplete or noisy measurements, which make classical numerical approaches difficult to scale or computationally expensive. By learning directly from data while remaining guided by the underlying physical trends, hybrid AI models can effectively handle such complexities, improve predictive accuracy, and provide robust estimates even when the governing processes are difficult to model using traditional deterministic solvers. As a result, AI-based algorithms offer a practical and flexible tool for modeling and forecasting coupled pollutant transport and aeration dynamics in real-world river systems.

Regarding model interpretability, it is important to note that since the input is restricted to the spatial coordinate (*x*), traditional feature importance analyses are not applicable. Instead, the model’s interpretability is established through its physical consistency; the RF-MLP framework successfully captures the complex, non-linear spatial gradients inherent in the Advection-Diffusion Equation, ensuring that the AI-driven predictions remain grounded in the deterministic laws of mass transport and longitudinal dispersion.

**Fig. 6:**
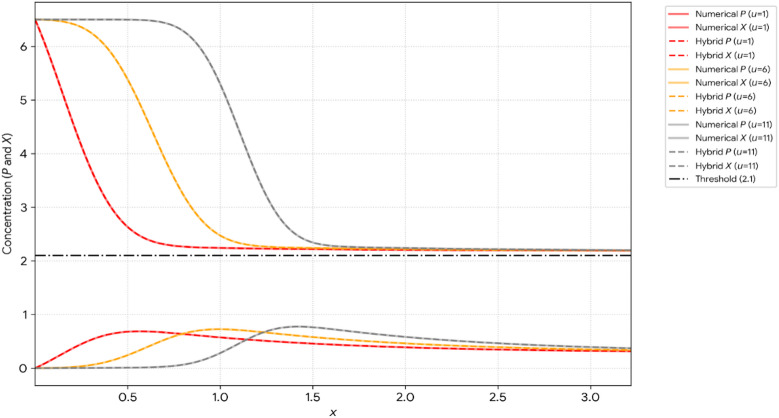
Predicted hybrid AI algorithm for the coupled water pollution and aeration concentrations vs. numerical solver actual data.

To demonstrate the framework’s generalizability beyond the analytical generating equation, the hybrid RF-MLP model was evaluated against an independent validation dataset generated via a Numerical Solver. As illustrated in Fig. 6, the hybrid RF-MLP algorithm demonstrates exceptional agreement with the numerical target, accurately capturing the peak concentrations and arrival times. It is worth mentioning that the cross-solver validation datasets generated via analytical solver (Fig. 5) as well as numerical solver (Fig. 6) provide rigorous evidence that the proposed hybrid RF-MLP algorithm has successfully internalized the underlying physical laws of the coupled pollution–aeration equations, rather than merely performing function approximation of the analytical formula. However, the authors emphasize that this cross-solver comparison fundamentally serves as a rigorous mathematical verification of physical model emulation and mathematical consistency. While it confirms that the hybrid RF-MLP architecture successfully emulates the continuous spatial gradients of the governing transport equations, it does not constitute site-specific empirical validation. Natural river systems exhibit complex, stochastic irregularities, transient boundaries, and sensor-level measurement noise that are simplified by idealized, deterministic differential equations. This cross-solver verification is therefore presented as an essential mathematical baseline, establishing physical consistency before deployment in natural real-world environments.

#### Physical based interpretation

In the numerical solution of Eqs. (9) and (10) using explicit finite difference method, the step length is assumed to be $\:\varDelta\:x=0.05\:\left(km\right)\:$ and $\:\varDelta\:t=0.002\:\left(day\right)$, for the ensuring of the stability of the finite difference scheme. The default parameter values 0 ≤ $\:x$ ≤ 4, $\:\mathrm{t}=0.1$ (day), $\:\mu\:=0.9\:\left(kg\:{km}^{-1}{day}^{-1}\right)$ $\:{\mathrm{D}}_{\mathrm{P}}=\:{\mathrm{D}}_{\mathrm{X}}=0.3{(\mathrm{k}\mathrm{m}}^{2}{\mathrm{d}\mathrm{a}\mathrm{y}}^{-1})$, $\:u$ = 1$\:\left({km\:day}^{-1}\right),$ $\:{\:K}_{1}=0.2$ $\:\left({day}^{-1}\right)$, $\:{\mathrm{K}}_{2}=1\left({day}^{-1}\right)$, $\:{\mathrm{K}}_{3}=0.3\left(\mathrm{k}\mathrm{g}{\:\mathrm{k}\mathrm{m}}^{-3}\right)$, $\:{\:K}_{4}={\:K}_{5}=1\left(km\right)$, $\:{\mathrm{P}}_{\mathrm{o}}=0.2,{\:\mathrm{P}}_{1}=0.8\:\left(\mathrm{k}\mathrm{g}{\:\mathrm{k}\mathrm{m}}^{-3}\right)$,$\:\:\:{\mathrm{X}}_{\mathrm{o}}=2.2\:\left(\mathrm{k}\mathrm{g}\:\mathrm{k}{\mathrm{m}}^{-3}\right)$, $\:{\mathrm{X}}_{1}=0.2\:\left(\mathrm{k}\mathrm{g}\:\mathrm{k}{\mathrm{m}}^{-3}\right)$,$\:\:{\mathrm{X}}_{2}=6.5\:\left(\mathrm{k}\mathrm{g}\:\mathrm{k}{\mathrm{m}}^{-3}\right)$, $\:{\uplambda\:}=0.8{(\mathrm{k}\mathrm{m}}^{-1})$, $\:{\upalpha\:}=0.01\:\left({\mathrm{d}\mathrm{a}\mathrm{y}}^{-1}\right)$ and $\:\mathrm{S}=7\:\left(\mathrm{k}\mathrm{g}\:{\mathrm{k}\mathrm{m}}^{-3}\right)$.

Figure 5 illustrates the spatial distributions of the dissolved oxygen concentration $\:X\left(x,t\right)$and the pollutant concentration $\:P\left(x,t\right)$ along the river at a fixed time for different values of the inlet flow velocity$\:\:\:u$, representing different rates of clean-water release at the upstream boundary. At the upstream location$\:\:x=0$, the dissolved oxygen concentration exhibits high values for all flow velocities due to the continuous injection of clean, oxygen-rich water. This reflects the implemented remediation strategy aimed at improving water quality in a river that was previously polluted as a result of past industrial activities. Since the injected water is free of pollutants, the pollutant concentration near the inlet remains very low, confirming the effectiveness of the clean-water flushing process in protecting the upstream region.

As the water flows downstream, the dissolved oxygen concentration gradually decreases due to its consumption by biochemical processes associated with the degradation of the existing pollutants, in addition to transport and dispersion effects. At the same time, the pollutant concentration initially increases, forming a characteristic downstream hump that represents the transport and redistribution of previously accumulated pollutants along the river. Further downstream, pollutant concentrations decrease due to dilution, dispersion, and degradation processes, indicating a gradual improvement in water quality.

The effect of the inlet flow velocity is clearly observed in both concentration profiles. For lower velocities, the residence time of water within the polluted reach is longer, which enhances oxygen consumption and results in a more rapid decline in dissolved oxygen levels. In contrast, higher flow velocities enhance longitudinal mixing and accelerate downstream transport, delaying the oxygen depletion and shifting the pollutant accumulation zone further downstream. Consequently, increasing the flow velocity improves the overall oxygen availability along the river while reducing localized pollutant buildup.

The horizontal dashed line represents 30% of the dissolved oxygen saturation concentration, which is adopted as the ecological threshold for sustaining aquatic life. The figure shows that, for the selected operating conditions, the dissolved oxygen concentration remains above this critical limit throughout the river reach for all considered flow velocities. This indicates that the applied clean-water release strategy is sufficient to maintain environmentally safe conditions along the river, allowing the ecosystem to support aquatic life.

From an environmental management perspective, the figure demonstrates how the proposed model can be used to assess the effectiveness of different remediation scenarios and to determine suitable flow velocities that ensure safe dissolved oxygen levels. By linking flow control with the coupled dynamics of pollutants and dissolved oxygen, the model provides a practical framework for identifying environmentally safe river sections that are suitable for fishing activities and fish farming, while also enabling the selection of the minimum clean-water release required to achieve these ecological objectives in a sustainable manner.

**Fig. 7:**
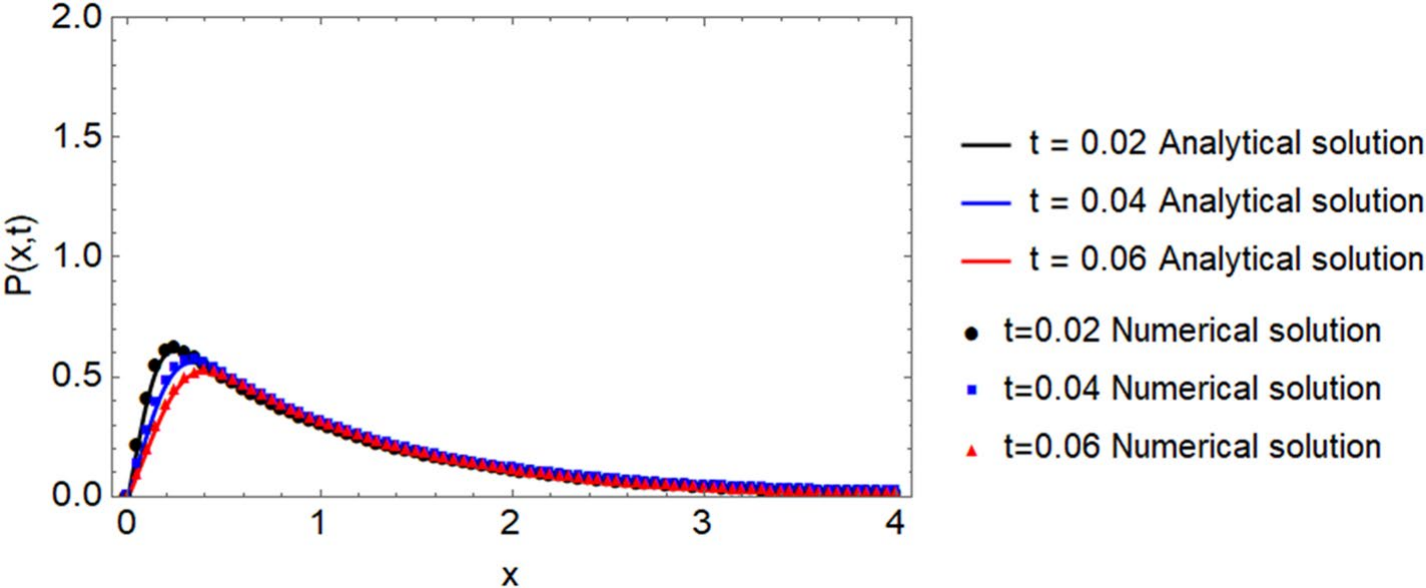
Comparison between the analytical and numerical solutions *P*(*x*,*t*).

**Fig. 8:**
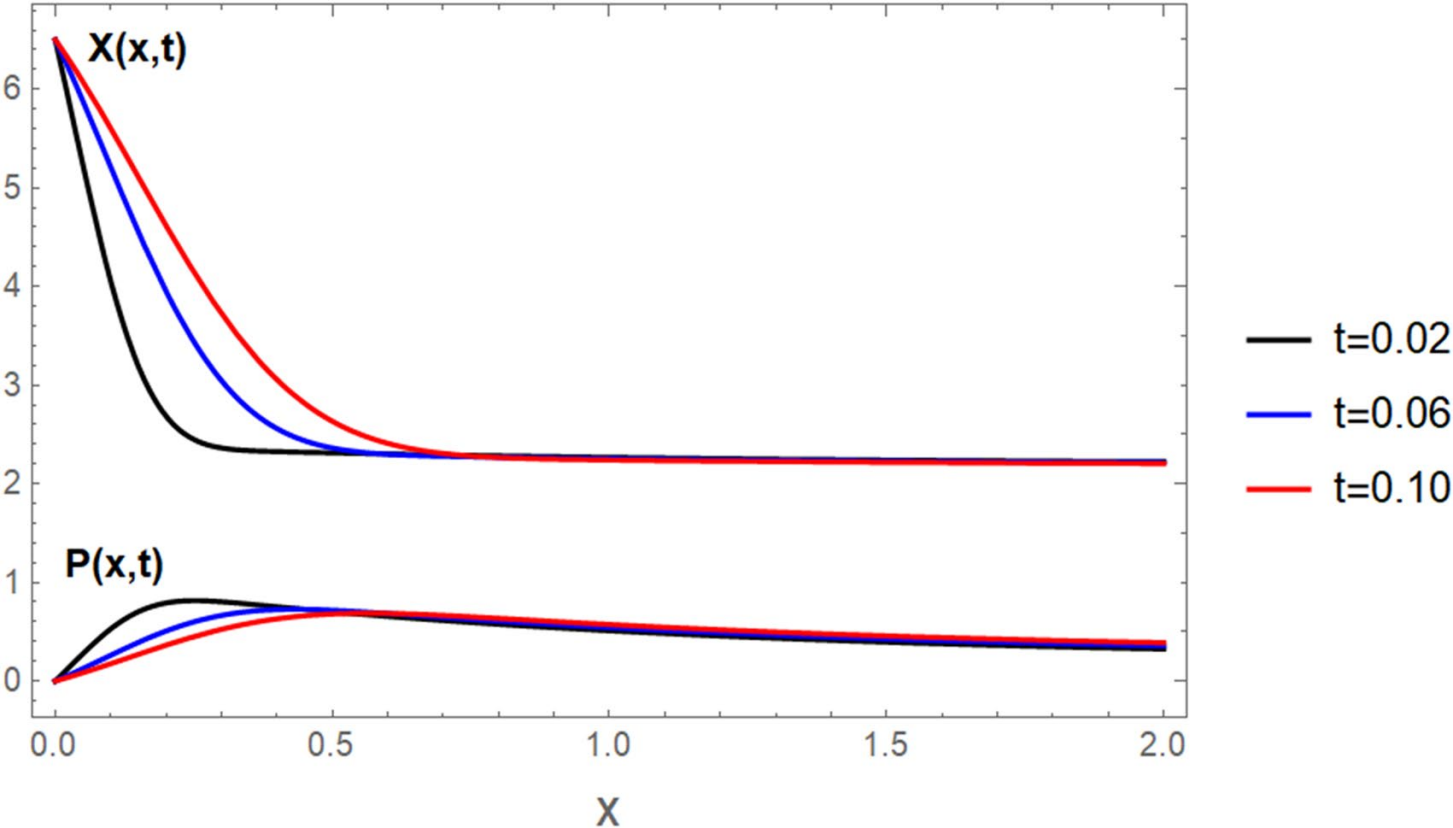
The variation of *P*(*x*,*T*) and *X*(*x*,*t*) with *t*.

**Fig. 9:**
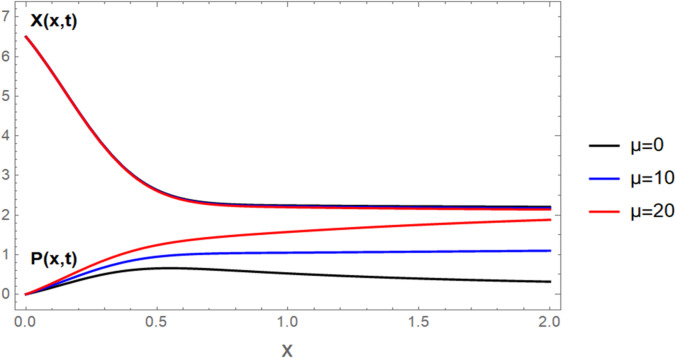
The variation of *P*(*x*,*T*) and *X*(*x*,*t*) with μ.

Figure 7 illustrates a comparison between the analytical solution given by Eq. (21) and the numerical solution of Eq. (9) computed using an explicit finite-difference method for $\:{K}_{1}={\upmu\:}={P}_{1}=0.$ The profiles are plotted at three different times, $\:\mathrm{t}=0.02,\:\:0.04,\:0.06.$ The results show excellent agreement between the two approaches across all tested times: the numerical results closely follow the analytical solutions, with only negligible differences attributable to discretization errors. This consistency may confirm the accuracy and stability of the explicit finite-difference scheme. Based on this validation, all subsequent results presented in Figs. 7, 8 and 9 are obtained using the explicit finite-difference method applied to the coupled advection–dispersion Eqs. (9) and (10).

Figure 8 presents the temporal evolution of pollutant concentration $\:P\left(x,t\right)$ and dissolved oxygen concentration $\:X\left(x,t\right)$ along the river at three different times, $\:\mathrm{t}=0.02,\:0.06,\:0.1$. Near the upstream boundary $\:x=0$, the dissolved oxygen concentration is high due to the injection of clean, oxygen-rich water. Moving downstream, the dissolved oxygen level gradually decreases as oxygen is consumed during pollutant degradation and transported by advection and dispersion. As time progresses, the influence of the injected clean water extends farther along the river, leading to a noticeable improvement in dissolved oxygen levels and a smoother spatial decay, which reflects the gradual recovery of the river system. In contrast, the pollutant concentration $\:P\left(x,t\right)\:$remains low near the upstream region because the injected water is free of pollutants. The concentration then increases to a maximum at a certain downstream distance as previously existing pollutants are transported and dispersed along the river. Farther downstream, the pollutant concentration decreases due to dilution and degradation processes. With increasing time, an overall reduction in pollutant concentration is observed throughout the river reach, indicating the effectiveness of the remediation strategy. Overall, the figure highlights the strong interaction between pollutant reduction and dissolved oxygen recovery, demonstrating a progressive improvement in water quality and the establishment of more favorable conditions for aquatic life.

Figure 9 illustrates the influence of the parameter $\:\mu\:$ on the spatial distributions of the pollutant concentration $\:P\left(x,t\right)$ and the dissolved oxygen concentration $\:X\left(x,t\right)$ along the river for $\:\mu\:=0,\:\:10,\:20$. It is observed from Fig. 8 that the dissolved oxygen curves are almost overlapping as the value of $\:\mu\:$ increases, indicating that the effect of $\:\mu\:$ on the spatial distribution of dissolved oxygen is relatively weak. This behavior can be attributed to the fact that the injected water at the upstream boundary contains a dissolved oxygen concentration close to saturation, which provides sufficient oxygen to compensate for the amount consumed during the water purification process, even with increasing $\:\mu\:$. In addition, natural reaeration contributes to maintaining nearly uniform dissolved oxygen levels along the river, thereby reducing its sensitivity to variations in $\:\mu\:$. In contrast, the pollutant concentration exhibits a clear response to increasing $\:\:\mu\:$, with higher values observed along the river as $\:\mu\:$ increases, particularly in the downstream region. This behavior reflects the direct role of $\:\mu\:$ in enhancing the presence of pollutants within the river system, compared to its limited influence on dissolved oxygen. Overall, the figure confirms that the clean-water injection strategy ensures the stability of dissolved oxygen levels, while the pollutant concentration remains more sensitive to increases in $\:\mu\:$.

**Fig. 10:**
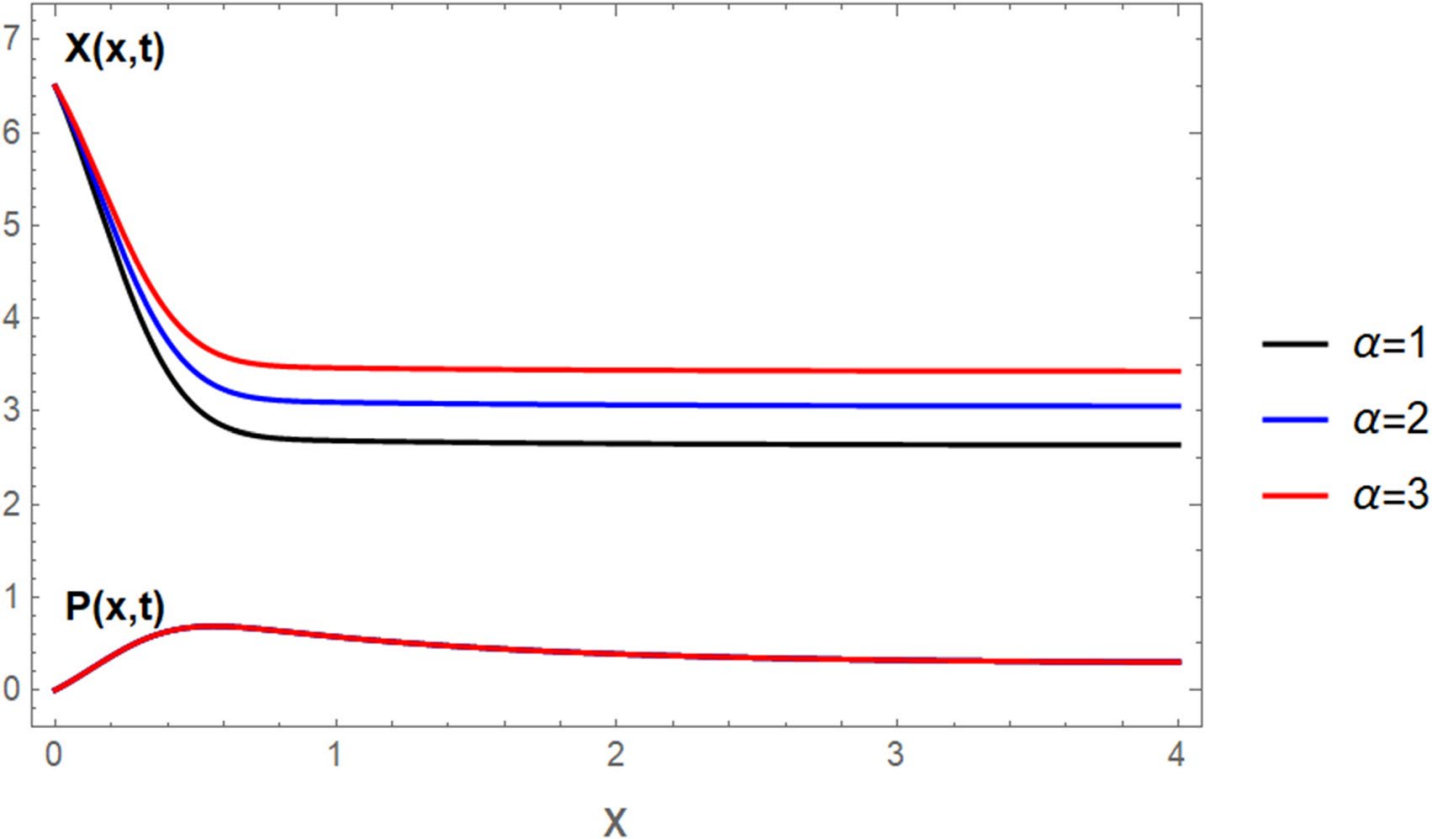
The variation of $\:P\left(x,T\right)\:and\:X(x,t)$ with $\:\alpha\:$.

Figure 10 illustrates the influence of the parameter $\:\alpha\:$ on the spatial distributions of the pollutant concentration $\:P\left(x,t\right)$ and the dissolved oxygen concentration $\:X\left(x,t\right)$ along the river for the values $\:\alpha\:=1,\:\:3,\:\:5$. The figure illustrates the effect of the reaeration coefficient $\:\alpha\:$ on the spatial distributions of the dissolved oxygen concentration $\:X\left(x,t\right)$ and the pollutant concentration $\:P\left(x,t\right)\:$along the river. It is observed that increasing the value of $\:\alpha\:$ leads to a noticeable increase in dissolved oxygen levels throughout the spatial domain, with higher $\:X\left(x,t\right)\:$curves corresponding to larger values of $\:\alpha\:$, particularly in the downstream regions. This behavior reflects the effective role of reaeration in replenishing the oxygen consumed during the water purification process and in maintaining higher and more stable dissolved oxygen levels within the river. In contrast, the pollutant concentration $\:P\left(x,t\right)\:$shows very similar profiles for different values of$\:\:\alpha\:$, indicating that the influence of reaeration on pollutant transport is relatively weak. This is because reaeration primarily affects the oxygen balance, while the pollutant dynamics are mainly governed by transport and degradation processes. Overall, the figure demonstrates that increasing $\:\alpha\:$ directly improves the environmental conditions by enhancing dissolved oxygen availability, while having a negligible effect on the spatial distribution of pollutants.

## Conclusion and future work

Water quality prediction and monitoring are essential for mitigating fish mortality and ensuring that commercial aquaculture operations remain both environmentally sustainable and economically viable. This study has successfully addressed these challenges by developing a novel physics-guided hybrid RF-MLP (residual) algorithm, trained on a high-fidelity dataset of 50,000 observations derived from the coupled pollution-aeration transport equations. The core conclusions of this research are summarized as follows:

- Methodological & Sustainability Contributions:
  - We established a reproducible framework integrating physical sciences with machine learning to identify optimal fish-survival zones (dissolved oxygen levels above 30% of saturation) along a polluted river reach.
  - By enabling decision-makers to determine the minimum allowable water velocity and upstream dissolved oxygen levels required to maintain safe conditions, this tool directly supports global sustainability initiatives, including United Nations Sustainable Development Goals for Clean Water and Sanitation (SDG 6) and Good Health and Well-Being (SDG 3).
- Computational & Statistical Performance:
  - The proposed hybrid RF-MLP architecture proved to be the superior predictive model, achieving an exceptionally high accuracy with an RMSE of 0.00012966 and an $\:{R}^{2}$ of 0.9999999692.
  - The sequential residual learning logic successfully combined the stable feature-partitioning of Random Forests (RF) with the continuous, non-linear error-correction of a Multilayer Perceptron (MLP), completely restoring the smooth physical gradients and eliminating the “step-like” artifacts typical of standalone tree-based models.
  - From an operational standpoint, the hybrid model offered an optimized performance-to-latency ratio, converging in just 22.58 s—representing a substantial 24.45 fold reduction in training duration compared to deep BiLSTM architectures (552.21 s) while maintaining superior accuracy over recurrent models (such as GRU and LSTM) and linear baselines.
- Physical & Hydrological Insights:
  - The physical-based analysis demonstrated that injecting clean, oxygen-rich water at the upstream boundary (*x = 0*) effectively dilutes pollutant concentrations near the inlet and progressively improves downstream dissolved oxygen levels over time.
  - Controlling the inlet flow velocity *u* is a critical lever for remediation; higher velocities enhance longitudinal mixing, accelerate downstream transport, and successfully delay the onset of oxygen depletion along the river channel.
  - Parametric evaluations revealed that while pollutant concentration remains highly sensitive to variations in the discharge source parameter $\:\mu\:$, the reaeration rate coefficient $\:\alpha\:$ exerts a dominant control on maintaining favorable downstream dissolved oxygen levels, whereas its influence on pollutant transport remains negligible.
  - Close agreement between the analytical solutions and the explicit finite-difference numerical scheme verified the mathematical stability and physical consistency of the underlying dataset used to guide the machine learning models.

The following recommendations for water resource managers and aquaculture planners are presented in order to empower future researchers to replicate the technique in diverse contexts and expand on the findings offered in this study:

1. Optimize Water Resources via Threshold-Based Flushing: Environmental managers should utilize the predictive model to identify and apply the minimum allowable upstream flushing velocity (u). Our spatial simulations indicate that while higher velocities accelerate pollutant dilution and delay downstream oxygen depletion, discharging excess clean water yields diminishing ecological returns. Identifying this optimal threshold ensures responsible water use while maintaining dissolved oxygen (DO) levels safely above the critical 30% ecological threshold required for fish survival.
2. Implement Dynamic Upstream Flow Control: To protect sensitive downstream fish farming zones, managers should dynamically adjust the upstream inlet velocity (u) in response to seasonal agricultural or industrial runoff cycles. Higher injection velocities successfully shift the “oxygen sag” (depletion) zone further downstream, effectively moving localized pollution away from critical aquaculture enclosures.
3. Prioritize Physical Reaeration Infrastructure: For long-term habitat restoration, physical remediation efforts (such as deploying cascade aerators, step structures, or modifying riverbed morphology to promote localized turbulence) must be prioritized. Our parametric evaluations reveal that the atmospheric reaeration rate coefficient ($\:\alpha\:$) exerts dominant, sensitive control over downstream oxygen recovery, while its overall impact on the transport of the pollutants themselves is negligible. Enhancing natural reaeration is therefore the most direct and sustainable way to maintain safe DO levels downstream.
4. Impose Strict Regulations on Point Sources: Because downstream pollutant concentration is highly sensitive to changes in the discharge source intensity parameter ($\:\mu\:$), regulatory bodies must strictly control active municipal and industrial discharge rates upstream. While clean-water flushing and natural reaeration are effective at preserving dissolved oxygen levels, they cannot fully neutralize massive spikes in upstream pollutant inputs.
5. Deploy Hybrid Physics-Guided AI for IoT Telemetry: For real-time water quality monitoring and early-warning alert networks, we strongly recommend adopting hybrid architectures like our sequential RF-MLP residual model over standalone deep learning models. Standalone recurrent networks like BiLSTM are computationally heavy, requiring extensive training times (552.21 s). In contrast, our hybrid model achieves superior predictive accuracy with a substantial 24.45-fold reduction in training duration (converging in just 22.58 s), making it exceptionally well-suited for edge computing, low-power telemetry sensors, and rapid online retraining.

**Future research will expand the dimensional and multivariable scope**:

- **Multi-Dimensional Modeling**: Extending the current 1D model to 2D and 3D scenarios. This expansion is essential to account for transverse and vertical mixing processes, which are critical in wider or deeper river reaches where pollutant concentrations ($\:P\:$) and dissolved oxygen levels ($\:X\:$) are not uniform across the cross-section.
- **Multivariate Indicators**: Moving beyond the coupled forecasting of $\:P\:$ and $\:X\:$ to simultaneously predict additional intercorrelated parameters such as pH, temperature, and nutrient concentrations (Nitrogen and Phosphorus). This holistic approach better reflects the complex biochemical interactions within aquatic ecosystems.
- **Algorithmic Optimization**: Exploring the use of Transformer-based architectures or Physics-Informed Neural Networks (PINNs). These advanced structures could further reduce the 16.76 s training overhead observed between the standalone Random Forest model (5.83 s) and the Hybrid model (22.58 s), while ensuring that the predictions remain strictly bounded by the physical laws of mass conservation.
- **Empirical validation and field-data benchmarking**: Future research will focus on the empirical validation of the RF-MLP framework through benchmarking against real-world hydrological datasets. A rigorous comparative analysis will be conducted to evaluate the model’s predictive performance on field-observed pollutant concentrations versus the results derived from deterministic ADE-generated synthetic data. This transition is critical for assessing the framework’s generalizability and its resilience to the stochastic complexities—such as sensor noise and environmental turbulence—that characterize natural river systems but are typically absent in idealized synthetic environments.

## Data Availability

The datasets used and/or analysed during the current study available from the corresponding author on reasonable request.
